# Expanding the Genetic Code with Lysine Aminoacylation

**DOI:** 10.1021/jacs.6c03157

**Published:** 2026-03-16

**Authors:** Xinyu Li, Qinglei Gan, Chenguang Fan

**Affiliations:** † Cell and Molecular Biology Program, 3341 University of Arkansas, Fayetteville, Arkansas 72701, United States; ‡ Department of Chemistry and Biochemistry, 3341 University of Arkansas, Fayetteville, Arkansas 72701, United States

## Abstract

Lysine aminoacylation is a newly discovered protein post-translational modification that is found in humans. However, few studies have been implemented to further investigate its function, possibly due to limited tools to produce target proteins with homogeneously aminoacylated lysine residues at specific sites. To achieve this goal, we applied the genetic code expansion strategy, engineered pyrrolysyl-tRNA synthetase, and established orthogonal translation systems for ten types of lysine aminoacylation compatible for both bacterial and mammalian cells. Because metabolic enzymes are preferred substrate proteins of lysine aminoacylation, we tested the effect of lysine aminoacylation on metabolic enzymes and demonstrated that lysine valylation and tyrosylation impaired pyruvate kinase and glucose-6-phosphate dehydrogenase activities, respectively. Further *in vivo* studies showed that lysine valylation of pyruvate kinase decreased the basal glycolytic rate in living human cells. In summary, this work provides a toolbox to study lysine aminoacylation.

## Graphical abstract

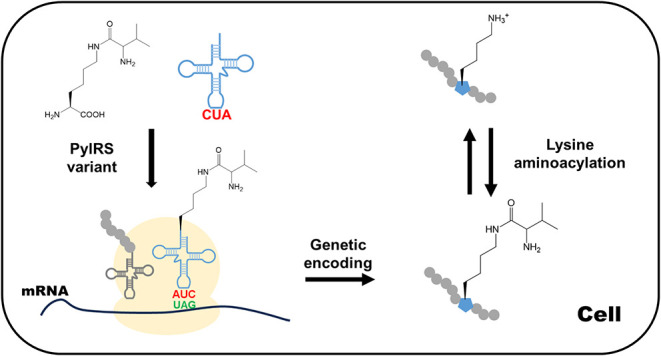

## Introduction

Lysine aminoacylation is an emerging post-translational modification of proteins, in which the ε-amine group of lysine is covalently linked to the α-carboxyl group of an amino acid.^1^ This modification was first observed in human liver cancer tissues by proteomic analyses.^1^ Approximately 3,500 proteins have been identified to have lysine residues that are aminoacylated by at least one of the 20 canonical amino acids. These substrate proteins are involved in a wide range of biological processes, with preferences for gene regulation, cell signaling, and metabolism, implying the role of lysine aminoacylation in sensing and transmitting intracellular amino acid signals.^1^ We further analyzed the data from the above-mentioned proteomic study for consensus sequences neighboring lysine aminoacylation sites (Figure 1). Interestingly, all 20 types of lysine aminoacylation have a strong preference for lysine or glutamate residues flanking aminoacylation-sensitive lysine residues.

**Figure 1:**
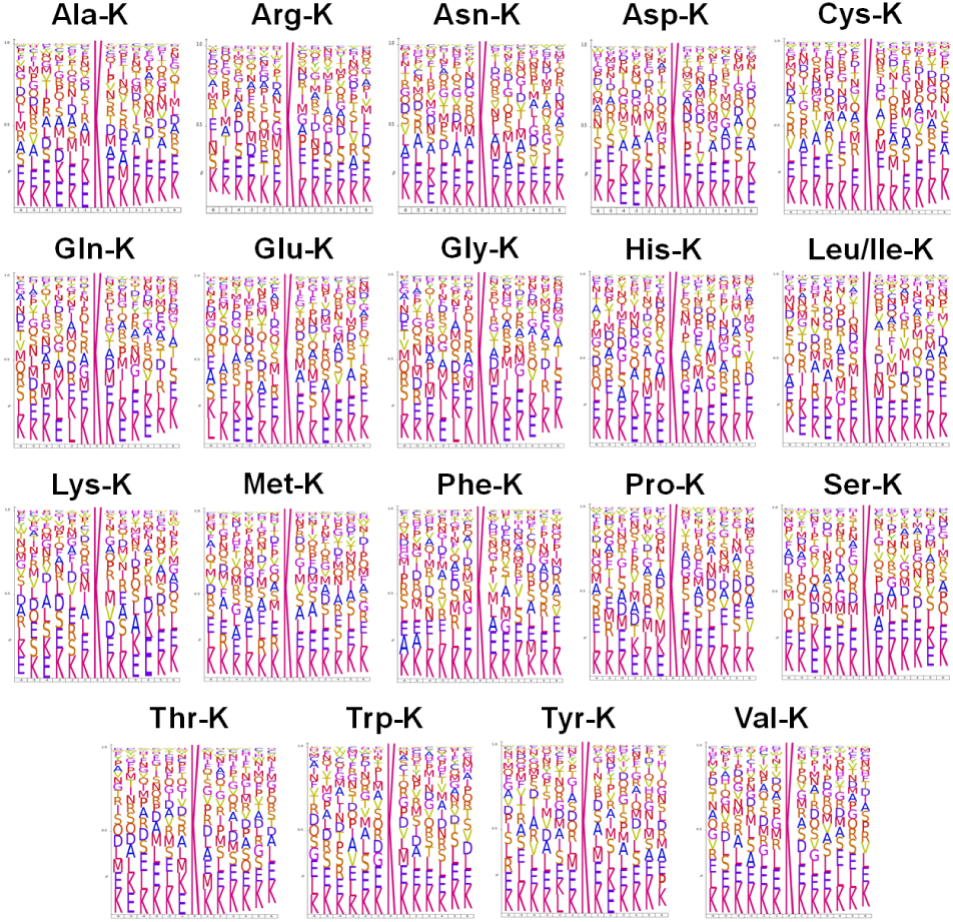
Consensus sequences of lysine aminoacylation sites. The consensus sequence analyses generated by IceLogo showed the amino acid composition at positions −6 to +6 relative to aminoacylation-sensitive lysine residues in substrate proteins of each type of lysine aminoacylation. The Y axis represents the probability of each amino acid in corresponding positions. Leu-K and Ile-K have the same molecular masses and cannot be distinguished by mass spectrometry; therefore, they were listed together.

The proposed mechanism of lysine aminoacylation is that the ε-amine group of the lysine residue is reacted with aminoacyl-AMP generated by corresponding aminoacyl-tRNA synthetase (AARS) to form a covalent bond with the α-carboxyl group of the amino acid^1^ (Figure 2a). This proposed mechanism was supported by *in vitro* aminoacylation assays with purified AARSs and target peptides as well as *in vivo* overexpression or inactivation of AARSs, which increased or decreased lysine aminoacylation levels correspondingly.^1^ The canonical function of AARSs is to activate cognate amino acids with ATP to form aminoacyl-AMP for tRNA aminoacylation.^2,3^ Aminoacyl-AMP is a high energy intermediate,^4^ and compounds containing such high-energy acyl-phosphate moieties are able to modify the ε-amine groups of lysine residues without enzymes.^5,6^ Although sporadic studies have reported that some AARSs can transfer aminoacyl groups to lysine residues in specific proteins, including lysyl-tRNA synthetase analogues for elongation factor P,^7,8^ methionyl-tRNA synthetase for itself,^9^ leucyl-tRNA synthetase for Ras-related GTP-binding protein A,^1^ and glutaminyl-tRNA synthetase for apoptosis signal-regulating kinase 1,^1^ it remains unclear whether lysine aminoacylation is an AARS-catalyzed reaction or not.

**Figure 2:**
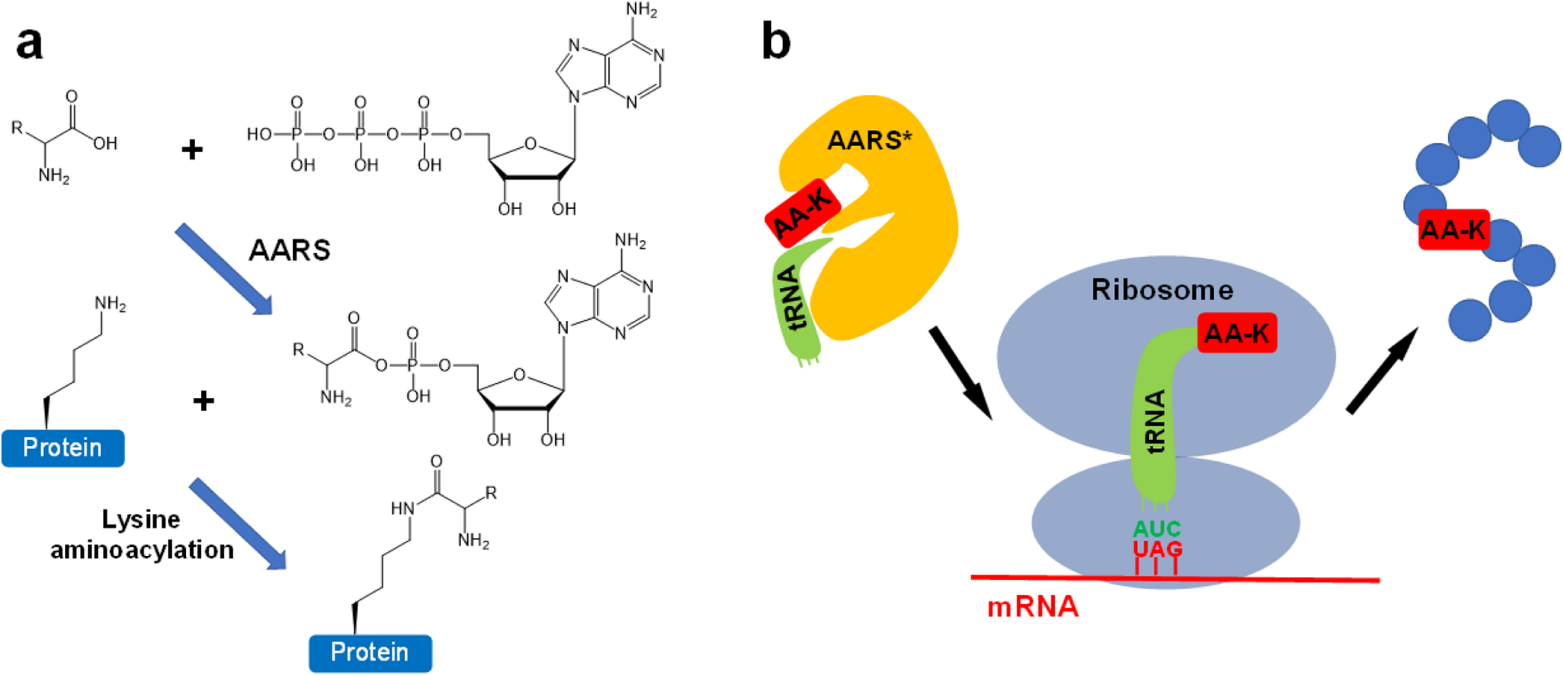
Schemes of lysine aminoacylation. a) Proposed mechanism of lysine aminoacylation in human cells. Aminoacyl-tRNA synthetase (AARS) activates its cognate amino acid with ATP to produce aminoacyl-AMP, which reacts with the ε-amino group of lysine residues in proteins to generate lysine aminoacylation. R represents the side chain of an amino acid. b) The scheme of genetic code expansion for lysine aminoacylation (AA-K) incorporation. AA-K is specifically recognized by an engineered AARS* and attached to an engineered tRNA, which decodes AA-K on the ribosome during translation in response to an introduced UAG stop codon, thus incorporating AA-K at a controlled site of the target protein.

To date, only few studies have been reported to further characterize lysine aminoacylation in specific proteins.^1,10^ One possible reason is that it is difficult to generate homogeneously aminoacylated proteins at target lysine sites due to the limited knowledge of substrate specificities. One study used glutamine as a mimic of lysine glutaminylation.^1^ However, glutamine is much smaller than authentic glutaminylated lysine, so it may not be a sufficient mimic for lysine aminoacylation. The most rigorous approach is to generate genuine lysine aminoacylation at the target sites. To achieve this goal, the genetic code expansion strategy was applied to generate authentic lysine threonylation in aurora kinase A to study its role in regulating cell cycle progression.^10^ The genetic code expansion strategy introduces a pair of an engineered AARS to recognize a modified amino acid (aminoacylated lysine in this study, AA-K) and an engineered tRNA that usually has a mutated anticodon to decode an assigned codon. Such pairs of AARS/tRNA, also called orthogonal translation systems (OTS), do not cross-react with native pairs of AARS/tRNA in host cells. Then, AA-K-charged tRNA reads the assigned codon (commonly a stop codon) in the mRNA to produce the target protein with AA-K at the controlled site^11,12^ (Figure 2b). To date, only three OTSs have been established to generate site-specific lysine aminoacylation in proteins, including lysine threonylation (Thr-K),^10,13^ lysine cysteinylation (Cys-K),^14^ and lysine methionylation (Met-K).^15^ Interestingly, the OTSs for Cys-K and Met-K were originally designed for studying lysine ubiquitination rather than lysine aminoacylation. In this work, we successfully developed OTSs for ten different types of lysine aminoacylation and investigated the effects of lysine aminoacylation on metabolic enzymes as well as their roles in regulating cellular metabolism in living human cells, providing a set of tools to study lysine aminoacylation, a newly discovered protein modification in humans.

## Results

### Establishing OTSs for Genetic Incorporation of Lysine Aminoacylation

To develop OTSs for lysine aminoacylation, we first evaluated existing OTSs for lysine modifications because of the similarity between the structures of lysine aminoacylation and those of certain lysine derivatives. Most of the OTSs for lysine modifications have been developed from the pair of pyrrolysyl-tRNA synthetase (PylRS) and tRNA^Pyl^ from *Methanosarcina* species because of its plasticity of the amino acid binding pocket and the orthogonality for both bacteria and eukaryotes.^12,16,17^ Furthermore, the structures of AA-Ks are similar to those of pyrrolysine, the native substrate of PylRS (Figure 3). Indeed, the existing OTSs for three AA-Ks (Thr-K, Cys-K, and Met-K) used either WT PylRS or PylRS variants for other lysine derivatives without further engineering.^10,13–15^ Thus, we first tested the recognition of WT *M. barkeri* PylRS (*Mb*PylRS) toward 20 types of AA-Ks by using the superfolder green fluorescent protein (sfGFP) as the reporter in *Escherichia coli* TOP10 cells. The codon for the permissive site Y151 in the sfGFP gene was mutated to a stop codon (TAG) by site-specific mutagenesis. The suppression of this stop codon with AA-K-charged tRNA^Pyl^ generated by WT *Mb*PylRS can produce full-length sfGFP to provide fluorescence readings. Our results showed that WT *Mb*PylRS only recognized Cys-K and Thr-K with suppression efficiencies of 20% and 25%, respectively (Figure 4a). Thus, further engineering was implemented to develop OTSs for other types of AA-Ks.

**Figure 3:**
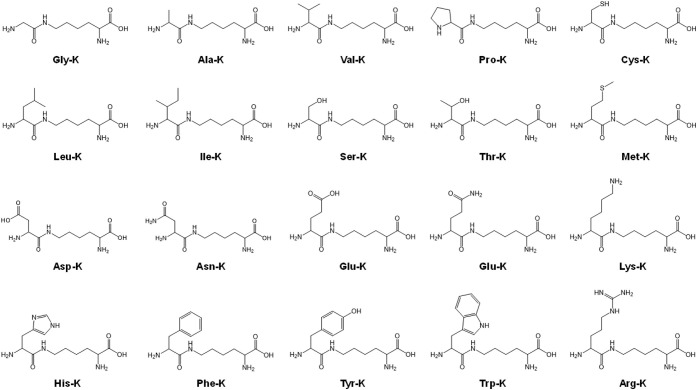
Structures of 20 types of aminoacylated lysine.

**Figure 4:**
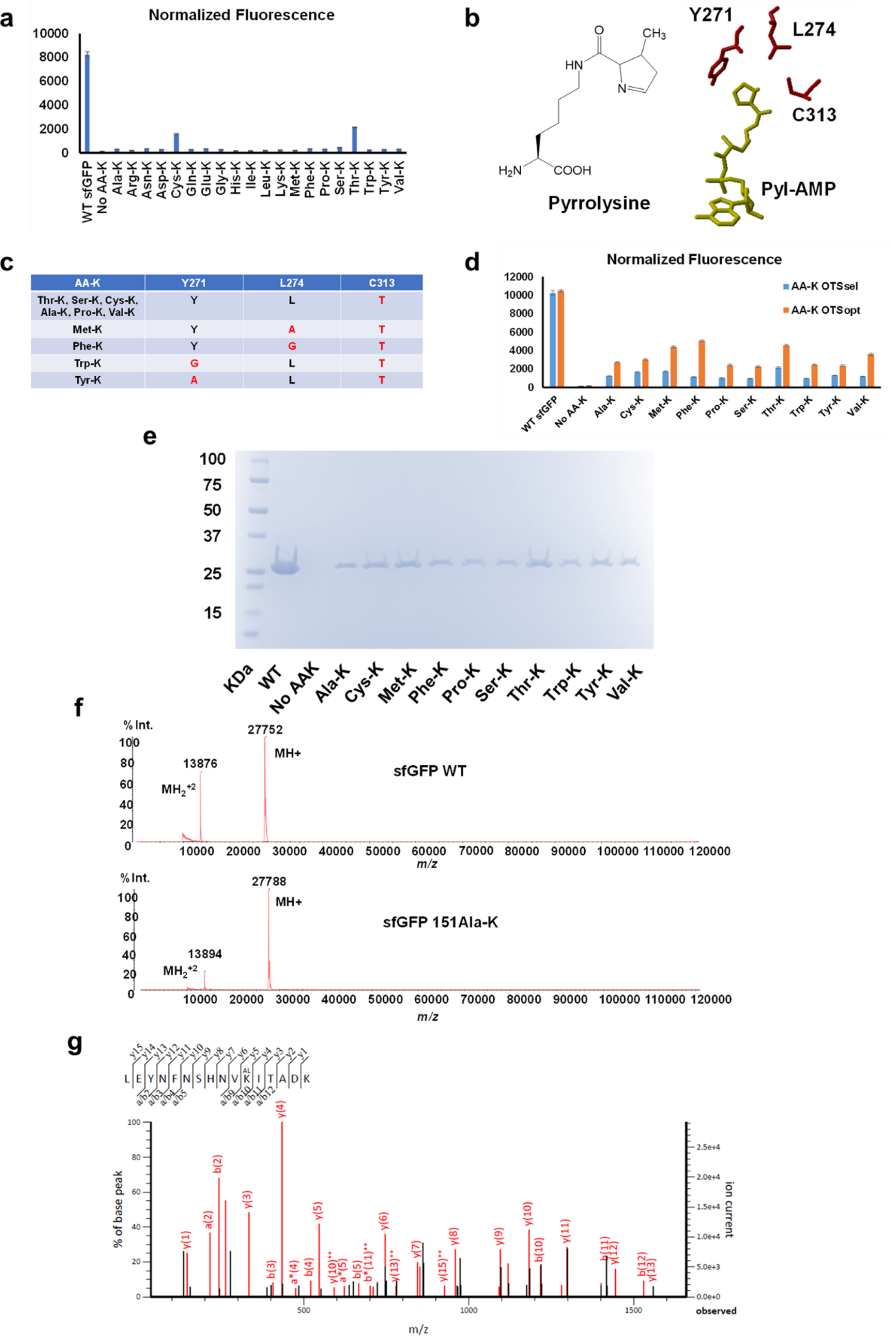
Development of the OTSs for lysine aminoacylation. a) Recognition of AA-Ks by WT PylRS based on sfGFP readthrough assays. The full-length WT sfGFP was used as the positive control to evaluate the suppression efficiency of the WT *Mb*PylRS and tRNA^Pyl^. The growth medium without any AA-Ks was used as the negative control to evaluate the baseline reading for the recognition of 20 canonical amino acids by WT *Mb*PylRS. The raw fluorescence reading was normalized by the cell density (OD_600_). b) Structures of pyrrolysine and the amino acid binding pocket of WT PylRS with Pyl-AMP (PDB ID: 2Q7H). c) The mutations in the PylRS variants for 10 types of lysine aminoacylation. Mutated residues were marked with red color. d) Comparison of OTSs derived from selected *Mb*PylRS variants (AA-K OTSsel) and optimized chPylRS variants (AA-K OTSopt) by the sfGFP readthrough assay. The raw fluorescence reading was normalized by the cell density (OD_600_). e) SDS-PAGE gel of purified WT sfGFP and its variants. Each lane was loaded with 1 μL of the elution fraction. f) The intact MS results for WT sfGFP and the 151Ala-K variant. The molecular weight difference is 36 Da, as expected (Ala-K replaces Tyr). Intact MS results for other sfGFP variants are in Figure S2–S10. g) The tandem MS result of the Ala-K-containing sfGFP fragment (L141-K156) by trypsin digestion of the purified sfGFP 151Ala-K variant. K^AL^ denotes Ala-K incorporation. The partial sequence of the peptide containing Ala-K can be read from the annotated a, b, or y ion series. Matched peaks are in red. The table of matching peaks is provided in Figure S11. Tandem MS results for other sfGFP variants are in Figure S12–S20.

According to the crystal structure of PylRS with its native substrate pyrrolysine (Figure 4b),^18^ we created a library of *Mb*PylRS variants with complete randomization of amino acids at positions Y271, L274, and C313. These three residues interact with the pyrroline ring of pyrrolysine, where the major structural variations of different AAKs occur. Then, we applied a selection strategy based on chloramphenicol resistance in the positive selection and CcdB (a DNA gyrase inhibitor) toxicity in the negative selection.^19,20^ Briefly, the plasmid containing the chloramphenicol acetyltransferase (CAT) gene with an internal stop codon at a permissive site (D112) was cotransformed into *E. coli* TOP10 cells with the plasmid harboring the library of PylRS variants in the positive selection. Any PylRS variants that can recognize AAK supplemented in growth media could read through the stop codon in the CAT gene to produce full-length CAT for cell survival against chloramphenicol. However, those PylRS variants that can recognize any canonical amino acids can also survive and should be removed. In the negative selection, AA-K was not provided in the growth media. The plasmid containing the CcdB gene with internal stop codons at two permissive sites (R13 and D44) was cotransformed into *E. coli* TOP10 cells with the plasmid pool of PylRS variants obtained in the positive selection. Those PylRS variants that can recognize any canonical amino acids could read through stop codons in the *ccdB* gene to produce full-length CcdB and were removed. We performed three cycles of positive and two cycles of negative selections alternatingly to increase OTS efficiency and orthogonality. Then, we used the sfGFP readthrough assay to evaluate the suppression efficiency of each PylRS variant obtained, and the best PylRS variants for 10 types of AA-Ks were listed (Figure 4c).

To further increase the efficiency of AA-K OTSs, we integrated outcomes from previous studies on optimizing PylRS-based OTSs. First, we adopted an optimized chimeric PylRS (chPylRS_opt_, comprising residues 1–149 of *Mb*PylRS and residues 185–454 of *M. mazei* PylRS with point mutations V31I, T56P, H62Y, and A100E, which showed enhanced efficiency without altering the substrate binding pocket of WT PylRS).^21^ The point mutation Y349F that had enhanced incorporation for lysine derivatives was also added.^22^ Then, we transplanted the mutations in *Mb*PylRS variants from selections to the corresponding positions in chPylRS_opt_ (Y271, L274, and C313). Compared with OTSs based on *Mb*PylRS variants from selections (AA-K OTS_sel_), chPylRS_opt_-based OTSs (AA-K OTS_opt_) increased fluorescence readings by 2–4 folds for all 10 types of lysine aminoacylation based on sfGFP readthrough assays (Figure 4d). Recently, PylRS from *Methanomethylophilus alvus* (*Ma*PylRS) has been widely used in the field of genetic code expansion for noncanonical amino acid (ncAA) incorporation.^23–26^ Thus, we transplanted the mutations in *Mb*PylRS variants from selections to the corresponding positions in *Ma*PylRS (Y126, M129, and V168) and added several mutations that could enhance incorporation efficiency (V42I, V75I, V168A, K181E, and A190V).^27^ However, there was no significant increase in *Ma*PylRS-based OTSs compared with chPylRS_opt_-based OTSs for AA-K incorporation by the sfGFP readthrough assay. Thus, we named those chPylRS_opt_ variants as corresponding AAKRSs through later studies.

To confirm the incorporation of AA-Ks at the correct positions, we used sfGFP as the reporter. The codon for the permissive site Y151 was mutated to TAG. For easy purification, we fused a His_6_-tag to the C-terminus of sfGFP. Only when the TAG stop codon is suppressed by the AA-K OTS, the full-length sfGFP with the C-terminal His_6_-tag can be generated and purified with affinity chromatography. If early termination occurs, then truncated sfGFP without the C-terminal His_6_-tag can be easily removed during the washing step during purification. We expressed and purified sfGFP variants with individual AA-K in *E. coli* BL21 (DE3) cells (Figure 4e). Nicotinamide was added in growth media to inhibit CobB deacetylase, which was shown to be able to remove lysine aminoacylation in cells.^1^ Previous studies have reported near-cognate suppression of the amber stop codon with canonical amino acids, including Trp, Tyr, Lys, Glu, and Gln.^28–30^ To determine the purity of the proteins, we first performed intact mass spectrometry (MS). All spectra showed high purity of sfGFP variants (Figures 4f and S1–S10). To further confirm the incorporation of AA-Ks at the correct position in sfGFP, we performed tandem MS after trypsin digestion (Figures 4g and S11–S20). We further analyzed the amino acid composition at position 151 (Table S1). Besides the expected AA-K incorporation, we also found lysine at the incorporation site in ∼5% of the total identified peptides containing position 151 for all 10 sfGFP variants. Besides near-cognate suppression, the appearance of lysine could also result from the process of trypsin digestion, which included pH changes and relatively long processing times, since we did not find other reported near-cognate amino acids (Trp, Tyr, Glu, or Gln) at the incorporation site. In summary, we obtained AA-K-containing sfGFP with ∼95% purity for all 10 types of AA-Ks.

### Lysine Aminoacylation Affects the Activities of Human Metabolic Enzymes

Besides protein synthesis, amino acids are also essential intermediates or precursors in metabolism.^31^ Metabolic enzymes have been identified as substrate proteins for all 20 types of lysine aminoacylation.^1^ Lysine modifications such as acetylation and succinylation, which have similar structures to lysine aminoacylation, are well-known for their roles in regulating cellular metabolism.^32,33^ Interestingly, most lysine aminoacylation sites in metabolic enzymes also undergo other lysine modifications, such as acetylation and succinylation,^34^ implying a potential role of lysine aminoacylation in metabolism.

To demonstrate the role of lysine aminoacylation in metabolism, we tested two essential metabolic enzymes associated with human diseases, which have been observed as substrate proteins of lysine aminoacylation by proteomic analyses.^1^ The first enzyme is pyruvate kinase M2 (PKM2) in the glycolysis pathway, which carries lysine valylation at K62 and K66. The other enzyme is glucose-6-phosphate dehydrogenase (G6PD) in the pentose phosphate pathway, which harbors lysine tyrosylation at K403. We expressed and purified these two enzymes with site-specific lysine valylation or tyrosylation by Val-K-OTS and Tyr-K-OTS, respectively. The codons at corresponding positions in the PKM2 and G6PD genes were mutated to the TAG stop codons. Again, we fused a His_6_-tag to the C-terminus of both proteins for easy purification. To eliminate the potential interference from the affinity tag, we expressed and purified WT enzymes with and without a C-terminal His_6_-tag, and there was no significant difference in enzyme activities between native and tagged proteins for both PKM2 and G6PD. Without AA-K supplemented in growth media, there were no visible bands by SDS-PAGE analyses for both proteins (Figure 5a and b). The yield of Val-K-containing PKM2 was ∼2 mg per liter culture, while the yield of Tyr-K-containing G6PD was ∼1 mg per liter culture. We first performed intact MS for purified PKM2 and G6PD as well as their variants, and all spectra showed high purity of proteins (Figures S21 and S22). We further confirmed the Val-K and Tyr-K incorporation at the correct sites in PKM2 and G6PD by tandem MS (Figures S23–S25). Similarly, we found only lysine but no other reported near-cognate amino acids at the incorporation sites in <10% of the total identified peptides containing the target sites (Tables S2 and S3). In summary, we obtained aminoacylated PKM2 and G6PD variants with >90% purity.

**Figure 5:**
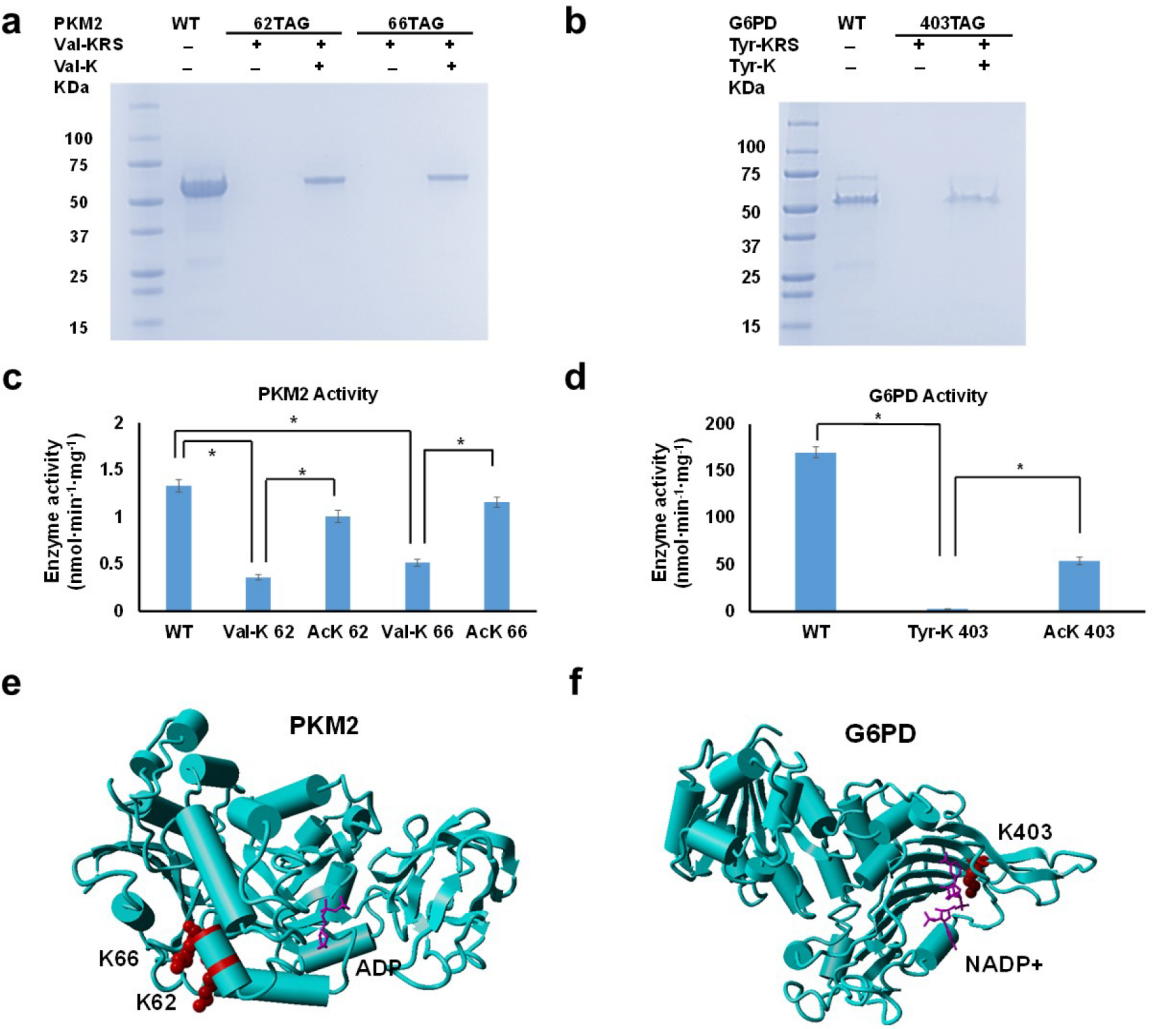
Effect of lysine aminoacylation on metabolic enzymes. a) SDS-PAGE gel of purified PKM2 and variants. b) The SDS-PAGE gel of purified G6PD and variants. For both gels, each lane was loaded with 5 μL of the elution fraction. c) Enzyme activity of purified PKM2 and variants. d) The enzyme activity of purified G6PD and variants. For both enzyme assays, 100 ng of purified enzyme was used in each assay. The values of mean and standard deviation were calculated based on three replicates. * indicates that the difference is significant at the level of 0.01. e) Crystal structure of human PKM2 (PDB ID: 3gr4). Lysine valylation sites were colored red, and the substrate ADP was colored with purple. f) The crystal structure of human G6PD (PDB ID: 6e08). The lysine tyrosylation site was colored red, and the substrate NADP+ was colored purple.

Next, we performed enzyme assays to determine the effect of lysine aminoacylation on PKM2 and G6PD (Figure 5c and d). For PKM2, valylation at K62 or K66 significantly decreased the enzyme activity, retaining only 27.2% and 38.7% of WT enzyme activity, respectively. Although we did not observe other canonical amino acids besides the original lysine at position 62 or 66 by MS analyses, we aimed to exclude the possibility that decreased enzyme activities resulted from the fidelity of the genetic code expansion strategy. Thus, we generated PKM2 variants with reported near-cognate amino acids Trp, Tyr, Glu, or Gln at position 62 or 66 individually (WT PKM2 has Lys at position 62 and 66). Enzyme assay results showed that all of these substitutions could not affect the enzyme activity significantly (Figure S26), indicating that lysine valylation is the major factor for decreased PKM2 activity. To further exclude the possibility that decreased enzyme activity was caused by misfolding of PKM2 variants with lysine valylation, we performed circular dichroism (CD) spectroscopy, which showed lysine valylation at K62 or K66 had no significant impact on the PKM2 structure (Figure S27). K62 and K66 are located near the entrance of the active site of PKM2, so valylation may affect the entry of the substrates (Figure 5e). Regarding G6PD, tyrosylation of K403 resulted in a complete loss of the enzyme activity. Again, we generated G6PD variants with Trp, Tyr, Glu, or Gln at position 403 (WT G6PD has Lys at position 403). Although the replacement with Trp or Tyr also decreased the enzyme activity significantly, both substitutions still retained ∼50% of the WT enzyme activity (Figure S28). Thus, lysine tyrosylation is the main reason for the complete loss of G6PD activity. CD spectra also demonstrated that lysine tyrosylation at K403 did not affect the G6PD structure significantly (Figure S29). K403 is located at the active site of G6PD and is close to NADP^+^, so adding a large tyrosyl group could interrupt the binding of substrates significantly (Figure 5f).

As mentioned above, different lysine modifications can occur at the same lysine site in proteins. Indeed, K62 and K66 of PKM2 as well as K403 of G6PD were also identified to be acetylated.^34^ To compare the impact of lysine acetylation and lysine aminoacylation on PKM2 and G6PD at those lysine sites, we expressed and purified PKM2 and G6PD with site-specific acetylation at the same lysine sites by the optimized OTS for acetyllysine incorporation^35^ and confirmed the incorporation of acetyllysine at correct sites by MS (Figures S30–32). Then, we measured the enzyme activities of acetylated PKM2 and G6PD variants (Figure 5c and d). Although lysine acetylation still affected both PKM2 and G6PD, their influences were significantly less than lysine aminoacylation, possibly due to size differences. Thus, lysine aminoacylation is a distinct modification from lysine acetylation regarding the impact on enzymes.

### Lysine Aminoacylation Impacts Cellular Metabolism in Living Human Cells

Since lysine aminoacylation was first discovered in human cells, we aimed to use human HEK293T cells as hosts to produce site-specifically aminoacylated proteins by the genetic code expansion strategy and to determine the effect of lysine aminoacylation on cellular metabolism in living human cells. Because of the orthogonality of the archaeal pair of PylRS/tRNA^Pyl^ in both bacterial and mammalian cells, PylRS-based OTSs have been successfully applied in HEK293T cells for ncAA incorporation by transforming mammalian cell-compatible vectors that carry the genes of PylRS variants and tRNA^Pyl^ originally engineered in *E. coli* cells.^36,37^

In *in vitro* studies mentioned above, we demonstrated that lysine valylation at K62 decreased PKM2 activity, while impaired PKM2 activity is one of the key features of cancer cells that facilitates tumor growth by shifting glucose metabolism from ATP production to nucleic acid and amino acid synthesis for rapid proliferation.^38^ Thus, we generated a PKM2 variant with lysine valylation at K62 in HEK293T cells by transforming a mammalian cell-compatible vector with the humanized Val-KRS gene and an optimized tRNA^Pyl^ for enhanced ncAA incorporation in mammalian cells.^39^ The K62 codon in the PKM2 gene was mutated to a TAG stop codon. We fused a 3X FLAG-tag to the C-terminus of PKM2 for easy identification and purification. To eliminate the potential interference from the affinity tag, we expressed and purified WT PKM2 with and without a C-terminal 3X FLAG-tag, and there was no significant difference in the enzyme activity between native and tagged PKM2. Without Val-K supplemented in growth media, there was no visible band by Western blotting with the 3X FLAG-tag antibody (Figure 6a). Then, we further confirmed the Val-K incorporation at position 62 in PKM2 by mass spectrometry (Figure 6b).

**Figure 6:**
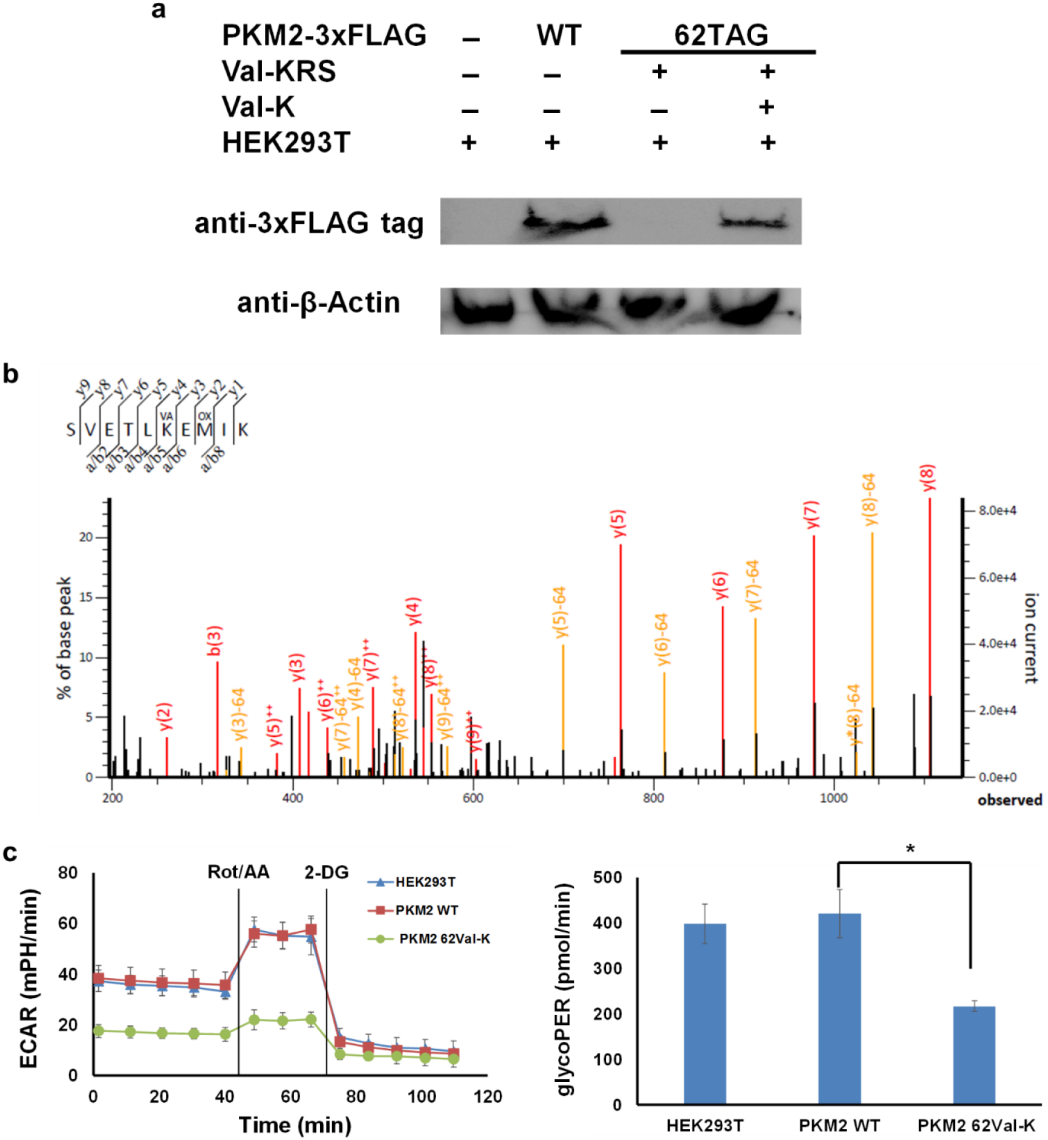
Impact of lysine valylation on cellular metabolism. a) Western blotting of HEK293 cells expressing PKM2 or its variant. The full images of Western blots are provided in Figure S33. b) Tandem mass spectrum of the Val-K-containing PKM2 fragment (S57–K66). K^VA^ denotes Val-K incorporation. M^OX^ denotes the oxidation of methionine. The table of matching peaks is provided in Figure S34. c) The left figure shows real-time extracellular acidification rates (ECARs). Rotenone/antimycin A (Rot/AA) and 2-deoxy-d-glucose (2-DG) were added at 42 and 67 min, respectively. The right figure shows the glycolytic proton efflux rate (glycoPER) as a measure of basal glycolysis (a physiological rate). Each cell line was tested with three replicates, and all values were normalized by cell numbers, with the cell number for HEK293T cells set as 1. * Indicates that the difference is significant at the level of 0.01.

PKM2 is the key enzyme in the glycolysis pathway; therefore, we performed the Seahorse XF glycolytic rate assay to measure the glycolytic rate in HEK293T cells expressing WT or the PKM2 variant with K62 valylated (Figure 6c). This glycolytic rate assay recorded basal measurements of the extracellular acidification rate (ECAR) from lactate generation, followed by sequential injection of rotenone/antimycin A to block mitochondrial activity, which removes the contribution of mitochondrial CO_2_ to ECAR and provides accurate measurement of glycolysis-linked ECAR. 2Deoxy-d-glucose was added later to inhibit glycolysis as an internal control.^40^ The glycolytic rate was correlated to ECAR. Clearly, cells expressing the valylated PKM2 variant had a significantly decreased glycolytic rate compared to cells expressing WT PKM2, consistent with enzyme assay results above, which showed lysine valylation of K62 decreased PKM2 activity.

In this study, we decided to keep endogenous PKM2 expressed in the genome for several reasons. First, as shown by Western blotting, the yield of valylated PKM2 was about one-third that of WT PKM2, so the difference in glycolytic rates could result from the difference in expression levels rather than lysine valylation alone if the native PKM2 gene is inactivated. Second, valylated PKM2 generated by the genetic code expansion strategy is valylated with high purity, while the physiological stoichiometry of lysine aminoacylation in cells ranges from 5% to 40%.^1^ Thus, the setup of mixing highly valylated PKM2 with native PKM2 better reflected real situations in cells better. As a control, we measured the glycolytic rate in HEK293T cells alone (Figure 6c). There was no significant difference between HEK293T cells and HEK293T cells expressing WT PKM2. This is possibly because PKM2 is not the speed-limiting step in glycolysis,^41^ and recombinant expression of PKM2 may not affect the overall glycolytic rate. On the other hand, the pool of PKM2 is a mixture of normal and impaired PKM2 in HEK293T cells expressing valylated PKM2, thus showing a decreased glycolytic rate.

## Discussion

In this work, we developed OTSs for 10 types of lysine aminoacylation. Among them, the OTSs for Thr-K, Cys-K, and Met-K had 2- to 5-fold improved incorporation efficiency compared with their previous OTSs based on WT PylRS or PylRS variants originally designed for other ncAAs. We have tried to select PylRS variants for the remaining 10 types of lysine aminoacylation, including His-K, Lys-K, Arg-K, Asp-K, Glu-K, Asn-K, Gln-K, Leu-K, Ile-K, and Gly-K. Based on the crystal structure of PylRS (Figure S35), we chose 3 additional sites (A267, L270, and W384) together with Y271, L274, and C313 to generate the library of PylRS variants and used the same selection approach. Unfortunately, we had no success in obtaining either PylRS variants for the remaining 10 types of lysine aminoacylation or better PylRS variants for the 10 existing types of lysine aminoacylation. His-K, Lys-K, Arg-K, Asp-K, and Glu-K are charged lysine derivatives that could have uptake issues. Proteins with site-specific incorporation of charged lysine derivatives such as lysine succinylation, malonylation, and glutarylation have been generated through genetic incorporation of ncAA precursors followed by further chemical conversion.^42^ In addition, several studies have utilized membrane transporters and pro-peptide strategies to overcome this problem.^43–45^ On the other hand, Asn-K and Gln-K contain the amide groups, which may need more hydrogen bonds for recognition. Leu-K and Ile-K are bulky, while Gly-K is relatively small. Thus, selection from the library of PylRS variants with more mutation sites is ongoing for developing the OTSs for them.

Besides developing OTSs for lysine aminoacylation, we also demonstrated the effect of lysine aminoacylation on essential metabolic enzymes and cellular metabolism both *in vitro* and *in vivo*. In addition to PKM2 and G6PD that are involved in carbohydrate metabolism, substrate proteins of lysine aminoacylation are also involved in the metabolism of lipids, amino acids, and nucleic acids (Table 1). Interestingly, we also noted that several AARSs are also substrate proteins of lysine aminoacylation, while AARSs are proposed to catalyze lysine aminoacylation themselves.^1^ Among them, lysyl-tRNA synthetase (KARS1) and threonyl-tRNA synthetase (TARS1) carry lysine residues aminoacylated by their cognate amino acids, while alanyl-tRNA synthetase (AARS1), aspartyl-tRNA synthetase (DARS2), and tryptophanyl-tRNA synthetase (WARS2) have lysine aminoacylation with noncognate amino acids. We further mapped those lysine sites onto their structures (Figure 7). K664 of AARS1 is located at the editing domain and undergoes leucylation or isoleucylation; therefore, the addition of a large hydrophobic group may change the conformation of the editing domain to affect translation fidelity. K578 (lysylation) of KARS1 and K164 (phenylalanylation) of DARS2 are located at tRNA binding regions; therefore, their aminoacylation could impair tRNA binding to affect translation efficiency. K199 of WARS2 is located at the dimer interface and undergoes phenylalanylation. The addition of an aromatic ring could impact dimer formation, which is necessary to form a complete catalytic pocket.^46^ K81 of TARS1 carries cognate threonine and is located at the TGS domain, which is involved in signaling and translation initiation.^47^ Thus, besides sensing amino acids for metabolism regulation, lysine aminoacylation could also play roles in translation or nontranslational functions related to AARSs, which could be a new mechanism of AARS regulation.

**Table 1:** Representative Metabolic Enzymes with Each Type of Lysine Aminoacylation

AA-K types	Representative metabolic enzymes	AA-K types	Representative metabolic enzymes
Ala-K	Enolase, Aconitase, ADP-sugar pyrophosphatase	Arg-K	Alcohol dehydrogenase, Glycogen synthase, Pyruvate dehydrogenase, Glucose-1-phosphate uridylyltransferase
Asn-K	Alcohol dehydrogenase, Fumarase, Pyruvate carboxylase, Aconitase, Pyruvate dehydrogenase, Acyl-CoA synthetase	Asp-K	Aldose reductase, Phosphoglycerate mutase, Hydroxymethylglutaryl-CoA lyase
Cys-K	Transaldolase, Methionine synthase, Acyl-coenzyme A synthetase	Gln-K	Glyceraldehyde-3-phosphate dehydrogenase, Triosephosphate isomerase,
Glu-K	Alcohol dehydrogenase, Aldolase, Succinate dehydrogenase,	Gly-K	Pyruvate dehydrogenase, Aldolase, Pyruvate kinase
His-K	Methionine synthase, Acetyl-CoA carboxylase,	Leu-K or Ile-K*	Alcohol dehydrogenase, Fatty acyl-CoA reductase, Aconitate, Acetyl-CoA synthetase
Lys-K	Alanine-glyoxylate aminotransferase, Inosine-5′-monophosphate dehydrogenase	Met-K	Glucose-6-phosphate translocase, Transketolase
Phe-K	Aspartate-tRNA synthetase, Tryptophan-tRNA synthetase	Pro-K	Alcohol dehydrogenase, Succinate synthase
Ser-K	Glyceraldehyde-3-phosphate dehydrogenase, Adenylate kinase	Thr-K	Threonine-tRNA synthetase, Acyl-CoA synthetase
Trp-K	Fructose-1,6-bisphosphatase, Acyl-CoA dehydrogenase	Tyr-K	Glucose-6-phosphate dehydrogenase, Enoyl-CoA hydratase,
Val-K	Pyruvate kinase, Hydroxymethylglutaryl-CoA synthase	*: Leu-K and Ile-K cannot be distinguished by mass spectrometry with the same molecular mass.	

**Figure 7:**
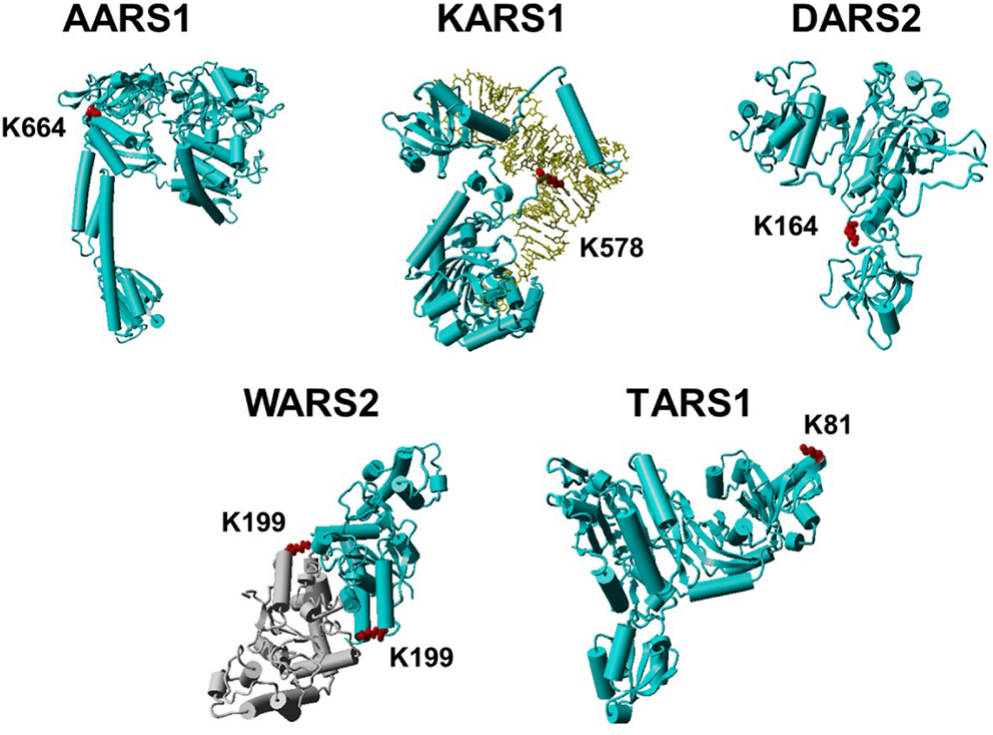
Structures of aminoacyl-tRNA synthetases with the sites of lysine aminoacylation. Aminoacyl-tRNA synthetases were colored blue or gray (if dimer), and tRNA was colored yellow (if any). Lysine residues undergoing aminoacylation were marked in red. There are no reported full-length structures for AARS1 and TARS1, so we used the structural models in the AlphaFold Protein Structure Database with model IDs AF-P49588-F1-v6 and AF-P26639-F1-v6, respectively. The PDB ID for KARS1 is 9DPL; the PDB ID for DARS2 is 4AH6; and the PDB ID for WARS2 is 5EKD. To better demonstrate that K164 of DARS2 is located at the tRNA binding region, we referred to the structure of *Pseudomonas aeruginosa* DARS with tRNA binding, which has a similar overall structure with human DARS2 as shown in Figure S36.

## Experimental Section

### Chemicals and Materials

Routine chemicals and materials for molecular biology and biochemical experiments such as growth media and antibiotics were purchased from Avantor (Radnor, PA, USA) or Sigma-Aldrich (St. Louis, MO, USA). Twenty types of AA-Ks were produced by liquid-phase peptide synthesis by Alan Scientific Inc. (Gaithersburg, MD, USA) following standard protocols. Briefly, the raw materials for individual amino acids that modify lysine were Boc-Gly-OH (CAS #: 4530-20-5), Boc-Ala-OH (CAS #: 15761-38-3), Boc-Cys­(Trt)-OH (CAS #: 21947-98-8), Boc-Pro-OH (CAS #: 15761-39-4), Boc-Asp­(OtBu)-OH (CAS #: 1676-90-0), Boc-Asn­(Trt)-OH (CAS #: 132388-68-2), Boc-Glu­(OtBu)-OH (CAS #: 13726-84-6), Boc-Gln­(Trt)-OH (CAS #: 132388-69-3), Boc-His­(Trt)-OH (CAS #: 32926-43-5), Boc-Val-OH (CAS #: 13734-41-3), Boc-Leu-OH (CAS #: 13139-15-6), Boc-Ile-OH (CAS #: 13139-16-7), Boc-Lys­(Boc)-OH (CAS #: 2483-46-7), Boc-Arg­(Pbf)-OH (CAS #: 200124-22-7), Boc-Phe-OH (CAS #: 13734-34-4), Boc-Tyr­(tBu)-OH (CAS #: 47375-34-8), Boc-Trp­(Boc)-OH (CAS #: 144599-95-1), Boc-Ser­(tBu)-OH (CAS #: 18942-50-2), Boc-Thr­(tBu)-OH (CAS #: 13734-40-2), and Boc-Met-OH (CAS #: 2488-15-5). Their carboxyl groups were activated and introduced to Boc-Lys-OH (CAS #: 13734-28-6). The desired purity (>90%) was achieved through purification and confirmed by HPLC and MS analyses. Other materials in specific experiments were mentioned in individual sections below.

### General Molecule Biology and Biochemical Experiments

Site-directed mutagenesis was implemented with the New England Biolabs (Ipswich, MA, USA) Q5 site-directed mutagenesis kit by following the manufacturer’s instruction. DNA sequences of all constructed plasmids were confirmed by whole plasmid sequencing by Plasmidsaurus Inc. (South San Francisco, CA, USA). Protein concentrations were determined by the Bradford protein assay. SDS-PAGE analyses were performed with 4–20% TGS precast protein gels and stained with Bio-Safe Coomassie stain purchased from Bio-Rad Laboratories (Hercules, CA, USA). For Western blotting, SDS-PAGE gels were first transferred onto PVDF membranes by a Trans-Blot Turbo Transfer System (Bio-Rad). After 2 h of blocking at room temperature with 5% bovine serum albumin and 0.1% Tween 20 in TBS buffer, transferred membranes were incubated overnight at 4 °C with 1:1000 diluted horseradish peroxidase (HRP)-conjugated antibodies purchased from Cell Signaling Technology (Danvers, MA, USA). On the second day, blotting membranes were visualized by chemiluminescence with a Pierce ECL Western blotting kit purchased from Thermo Scientific (Waltham, MA, USA).

### The Superfolder GFP (sfGFP) Readthrough Assay

The sfGFP gene (WT or Y151TAG mutant) was inserted into the *pBAD* plasmid under the control of the arabinose promoter. The genes of PylRS (or variants) and tRNA^Pyl^ were cloned into the *pTech* plasmid under the control of the pLpp promoter and the proK promoter, respectively. Both plasmids were transformed into *E. coli* TOP10 cells (Thermo Fisher Scientific). Cells were then inoculated into 2 mL of LB medium and grown at 37 °C overnight. On the second day, the overnight culture was diluted to 200 μL in a well of a 96-well plate with fresh LB medium to an absorbance at 600 nm (OD600) of 0.15. The growth medium was supplemented with 5 mM AAK individually. The expression of sfGFP was induced by adding 1 mM arabinose. The 96-well plate was then shaken continuously for 12 h at 37 °C. The fluorescence intensity (excitation 485 nm, emission 528 nm, bandwidths 20 nm) and OD600 of each well were recorded hourly by a BioTek microplate reader (Winooski, VT, USA).

### PylRS Variant Library Construction and Selection for AA-K-Specific Variants

For PylRS variant library construction, three residues (Y271, L274, and C313) of PylRS were randomly mutagenized with two primers (PylRS271/273-QF: GCACCGAACCTG **NNN** AATTAC- **NNN** CGTAAACTGGATC and PylRS313-QF: CATGCTGAATTTC **NNN** CAAATGGGCTCG) with the QuikChange Multisite-Directed Mutagenesis kit (Agilent Technologies, Santa Clara, CA, USA). For the positive selection, 50 ng of the pBK-PylRS library plasmid was introduced into 50 μL *E. coli* TOP10 (∼10^8^ cells) with the positive selection plasmid pCAT-pylT, which carries the tRNA^Pyl^ gene and a mutant *cat* gene with an amber stop codon TAG at corresponding position of D112 in CAT. After recovered in 1 mL of SOC at 37 °C for 2 h, cells were added into 100 mL of fresh LB medium and grown overnight at 37 °C. On the next day, 100 μL of the overnight culture was inoculated into 5 mL of fresh LB medium with 5 mM AA-K individually. After growing at 37 °C for 4 h, 200 μL of the culture (∼5 × 10^7^ cells) was plated on the LB plate with 5 mM individual AA-K and 50 μg/mL chloramphenicol. The plates were then incubated at 37 °C for 48 h, and all the colonies growing on the plate for each AA-K were scraped and resuspended in 5 mL of fresh LB media and grown for another 4 h at 37 °C, individually. Then, total plasmids were extracted by the Qiagen plasmid purification kit (Hilden, Germany), and the pBK-PylRS library plasmids were isolated by agarose gel electrophoresis and purified by the Promega gel purification kit (Madison, WI, USA). For the negative selection, 50 ng of pBK-PylRS library plasmids from the positive selection for each AA-K were transformed into 50 μL *E. coli* TOP10 individually with the negative selection plasmid pAraCB2-pylT, which carries the gene of tRNA^Pyl^ and a mutant *ccdB* gene with two amber stop codons at positions 13 and 44. The transformants were recovered in 1 mL of SOC at 37 °C for 2 h, and then, 100 μL of the culture was plated on an LB plate with 0.2% arabinose but without any AA-K. After incubation at 37 °C overnight, all the colonies were harvested, and pBK-PylRS library plasmids for each AA-K were extracted and isolated separately using the same procedures as the positive selection. Then, the pBK-PylRS library for each AA-K underwent positive–negative-positive selection to increase the OTS efficiency and orthogonality. The chloramphenicol concentration was increased to 100 and 150 μg/mL in the second and third rounds of positive selection, respectively. Finally, the pBK-PylRS plasmids extracted and isolated from each single clone were sent for DNA sequencing by Eurofins Genomics (Louisville, KY, USA) with two primers: pBK-F: gcagagcattacgctg-acttgacgggacgg and pBK-R: ctgtttcttgccggatgcggcgtgaacgcc.

### Expression and Purification of His-Tagged Proteins in *E. coli* Cells

The gene of the target protein was cloned into the *pCDF* plasmid with a C-terminal His_6_-tag, which was then transformed into BL21­(DE3) cells together with the *pTech* plasmid carrying the genes of tRNA^Pyl^ and AA-KRS. The expression strain was grown in 400 mL of LB medium supplemented with 5 mM AA-K at 37 °C to OD600 of 0.6–0.8. After the addition of 0.1 mM IPTG to induce protein expression and 20 mM nicotinamide to inhibit deacetylase, cells were then incubated at 30 °C for 6 h and harvested by centrifugation at 5000 × g for 15 min at 4 °C. The cell pellet was resuspended in 10 mL of 50 mM Tris pH 7.5, 300 mM NaCl, 20 mM imidazole (lysis buffer) with Roche protease inhibitor cocktail (Basel, Switzerland) and broken by sonication. The crude extract was centrifuged at 20,000 × g for 30 min at 4 °C. The soluble fraction was filtered with a 0.45 μm filter and loaded onto a column containing 1 mL of Ni-NTA resin (Qiagen), which was equilibrated with 20 mL of lysis buffer. The column was then washed with 30 mL of 50 mM Tris pH 7.5, 300 mM NaCl, and 50 mM imidazole, and the target protein was eluted with 2 mL of 50 mM Tris pH 7.5, 300 mM NaCl, 200 mM imidazole. The elution fraction was then desalted by the PD-10 column (GE Healthcare, Chicago, IL, USA) with desalting buffer 50 mM Tris pH 7.5, 20 mM NaCl.

### Intact Mass Spectrometry (MS) Analyses

Intact MS analyses were performed at the University of Arkansas Statewide Mass Spectrometry Facility. Purified enzymes or their variants were diluted to 0.1 mg/mL and desalted using the ZipIip protocol. Desalted protein samples were vacuum-evaporated, mixed with the Sinapic acid MALDI matrix, and analyzed by a Shimadzu AXIMA MALDI-TOF mass spectrometer. Spectra were obtained in the positive ion linear mode.

### LC–MS/MS Analyses

The target protein band at the corresponding molecular weight of the SDS-PAGE gel was cut and sent to the proteomics facility of the Yale Keck Biotechnology Resource Laboratory for MS analyses. Proteins were trypsin-digested by a standard in-gel digestion protocol and analyzed by LC–MS/MS on an LTQ Orbitrap XL instrument (Thermo Scientific) with a nanoACQUITY UPLC system (Waters). Peptides were separated by a Symmetry C18 trap column and a nanoACQUITY UPLC column. Trapping was performed at 15 μL/min and 99% buffer A (0.1% formic acid) for 1 min, and peptide separation was implemented at 300 nL/min with buffer A and buffer B (CH_3_CN in 0.1% formic acid). The linear gradient was from 5% buffer B to 50% buffer B at 50 min, and to 85% buffer B at 51 min. MS data were acquired in the Orbitrap with one microscan and a maximum inject time of 900 ms, followed by data-dependent MS/MS acquisitions in the ion trap (through collision-induced dissociation). The Mascot search algorithm was used to search for the AA-K substitution (Matrix Science, Boston, MA, USA).

### Enzyme Assays for PKM2 and G6PD

The activities of WT PKM2 and its variants were measured with the commercially available pyruvate kinase assay kit from BioAssay Systems (Hayward, CA, USA). In this assay, PEP and ADP are catalyzed by pyruvate kinase to generate pyruvate and ATP. The color intensity of the reaction product at 570 nm is directly proportional to the pyruvate generated by PKM2. 100 ng portion of PKM2 or its variants was used for each assay. The activities of WT G6PD and its variants were measured with the commercially available glucose-6-phosphate dehydrogenase assay kit from BioAssay Systems. This assay is based on the reduction of the tetrazolium salt MTT in an NADPH-coupled enzymatic reaction to a reduced form of MTT that exhibits an absorption maximum at 565 nm. The increase in absorbance at 565 nm is proportional to G6PD activity. 100 ng of G6PD or its variant was used for each assay. Both assays were performed by following the manufacturer’s protocols in 96-well plates and read by the microplate reader. For each sample, three replicates were tested to calculate the mean and standard deviation.

### Circular Dichroism (CD) Spectrometry

The CD spectrometry analysis was performed by using a J-1500 CD Spectrometer (JASCO Corporation, Tokyo, Japan). Purified enzymes or their variants were diluted to a concentration of 0.1 mg/mL in 5 mM Tris-HCl pH 7.8, 0.1 M KCl, and scanned from 190 to 250 nm at a 60 nm/min speed. Scanning was performed five times for each sample, and the average was plotted.

### Expression of 3X FLAG-Tagged PKM2 in HEK293T Cells

HEK293T cells and growth media were purchased from ATCC (Manassas, VA, USA). HEK293T cells were maintained in Dulbecco’s Modified Eagle’s Medium (DMEM) supplemented with 10% Fetal Bovine Serum (heat-inactivated), 2 mM l-glutamine, and 1% penicillin-streptomycin. Cells were incubated in a humidified chamber at 37 °C with 5% CO_2_. The plasmid containing Val-KRS was modified from pNEU-hMbPylRS-4xU6M15 purchased from Addgene (Watertown, MA, USA). The humanized Val-KRS gene was cloned to replace the hMbPylRS gene and confirmed with whole plasmid sequencing. The PKM2 gene was cloned into pcDNA3.1-Hygro (GenScript, Piscataway, NJ, USA) and confirmed with whole plasmid sequencing. About 3 × 10^5^ HEK293T cells were seeded in a 6-well plate with 2 mL of complete growth media to ∼80% confluency. Two mM ValK was added to growth media 2 h before transfection. The ATCC TransfeX reagent (5 μL) was used to transfect 293T cells with 2 μg pNEU-hValKRS-4xU6M15 and 2 μg pcDNA3.1-Hygro-PKM2-3xFLAG in 250 μL Opti-MEM. After 2 days, cells were collected, washed with PBS, and transferred to T75 flasks with fresh growth media with 2 mM Val-K to 60–70% confluency. For protein purification, ∼10^7^ cells were collected and lysed by the cell lysis buffer with protease inhibitor cocktails purchased from Cell Signaling Technology. The crude extract was centrifuged at 14,000*g* for 10 min. The supernatant was filtered with a 0.45 μm filter, and 3X FLAG-tagged PKM2 was purified with anti-FLAG tag affinity beads by following the manufacturer’s instruction (Abcam, Cambridge, UK). Briefly, beads were washed 3 times with 50 mM Tris, 0.15 M NaCl, pH 7.4, and PKM2 was eluted with 50 mM Tris, 0.15 M NaCl, pH 7.4 with 100 μg/mL 3X FLAG competitive peptide (Sigma-Aldrich).

### The Seahorse XF Glycolytic Rate Assay

The glycolytic rate assay was performed by an Agilent Seahorse XFe24 Extracellular Flux Analyzer (Agilent Technologies), which directly measures the real-time extracellular acidification rate (ECAR) or oxygen consumption rate (OCR) of cells to determine the glycolytic proton efflux rate (glycoPER) of the cells. A sensor cartridge was hydrated in Seahorse XF Calibrant at 37 °C overnight in a non-CO_2_ cell incubator. On the day prior to the assay, transfected HEK293T cells were seeded into a 24 well-plate at cell densities of 80K cells/well using the DMEM growth medium. XF assay media were prepared by supplementing DMEM with 1 mM pyruvate, 2 mM glutamine, and 10 mM glucose. Before experiments, cell culture media were replaced with 500 μL of fresh XF assay media. For the glycolytic rate assay, 5 μM rotenone/antimycin A (Rot/AA) and 500 mM 2-deoxy-d-glucose (2-DG) were added into ports at 42 and 67 min, separately. For basal measurement: 5 cycles with 3 min of mixing, 3 min of wait/equilibration time, and 3 min of measurement per cycle. Injection 1 (port A) Rot/AA with 3 cycles with 3 min of mixing, 2 min of wait/equilibration time, and 3 min of measurement per cycle. Injection 2 (port B) 2-DG with 5 cycles with 3 min of mixing, 2 min of wait/equilibration time, and 3 min of measurement per cycle. Data analyses were processed by using Agilent Seahorse Analytics software.

## Supplementary Material









## Notes

Full peptide results of trypsin digestion for sfGFP variants (Table S1) (XLSX)
Full peptide results of trypsin digestion for PKM2 variants (Table S2) (XLSX)
Full peptide results of trypsin digestion for the G6PD variant (Table S3) (XLSX)
Intact mass spectra of AA-K-containing sfGFP (Figures S1–S10); tandem mass spectra of AA-K-containing sfGFP (Figures S11–S20); intact mass spectra of WT PKM2 and its variants (Figure S21); intact mass spectra of WT G6PD and its variant (Figure S22); tandem mass spectra of AA-K-containing PKM2 or G6PD (Figures S23–S25); enzyme activities of PKM2 variant controls (Figure S26); CD spectra of PKM2 and its variants (Figure S27); enzyme activities of G6PD variant controls (Figure S28); CD spectra of G6PD and its variant (Figure S29); tandem mass spectra of acetyllysine-containing PKM2 or G6PD (Figures S30–S32); full images of Western blotting for PKM2 expressed in 293T cells (Figure S33); the tandem mass spectrum of Val-K-containing PKM2 expressed in 293T cells (Figure S34); the structure of PylRS and additional mutation sites (Figure S35); the structure of *Pseudomonas aeruginosa* aspartyl-tRNA synthetase with tRNA (Figure S36) (PDF)
