# Supplementary material for: Expanding the Genetic Code with Lysine Aminoacylation
Source: J Am Chem Soc. 2026 Mar 16;148(11):12411–22. doi: 10.1021/jacs.6c03157 (PMC13022859; doi:10.1021/jacs.6c03157)

Supporting Information

Expanding the genetic code with lysine aminoacylation

Xinyu Li, Qinglei Gan, Chenguang Fan*

Table of Content

Figures S1-10. Intact mass spectra of AA-K-containing sfGFP.

Figure S11-20. Tandem mass spectra of AA-K-containing sfGFP.

Figure S21. Intact mass spectra of WT PKM2 and its variants.

Figure S22. Intact mass spectra of WT G6PD and its variant.

Figures S23-S25. Tandem mass spectra of AA-K-containing PKM2 or G6PD.

Figure S26. Enzyme activities of PKM2 variant controls.

Figure S27. CD spectra of PKM2 and its variants.

Figure S28. Enzyme activities of G6PD variant controls.

Figure S29. CD spectra of G6PD and its variant.

Figures S30-S32. Tandem mass spectra of acetyllysine-containing PKM2 or G6PD.

Figure S33. Full images of western blotting for PKM2 expressed in 293T cells.

Figure S34. The tandem mass spectrum of Val-K-containing PKM2 expressed in 293T cells.

Figure S35. The structure of PylRS and additional mutation sites.

Figure S36. The structure of *Pseudomonas aeruginosa* aspartyl-tRNA synthetase with tRNA.

Table S1. Full peptide results of trypsin digestion for sfGFP variants as a separate Excel file.

Table S2. Full peptide results of trypsin digestion for PKM2 variants as a separate Excel file.

Table S3. Full peptide results of trypsin digestion for the G6PD variant as a separate Excel file.

Figure S1. The MALDI-TOF analysis of purified sfGFP-151Ala-K variant. The expected molecular weight is 27784 Da.

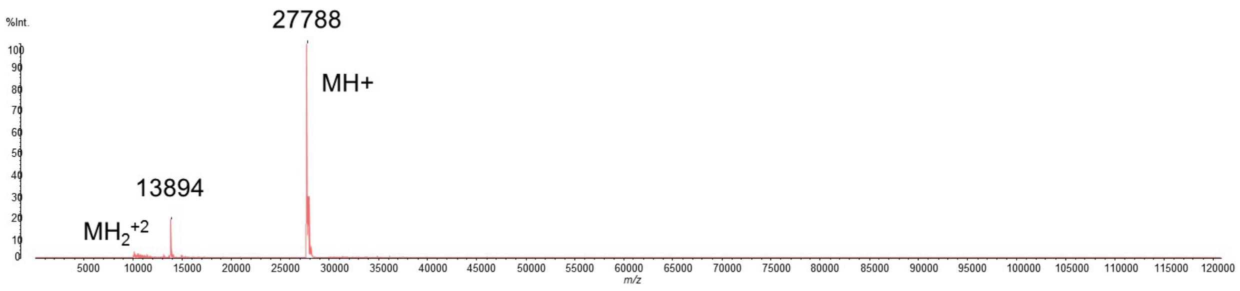

Figure S2. The MALDI-TOF analysis of purified sfGFP-151Cys-K variant. The expected molecular weight is 27816 Da.

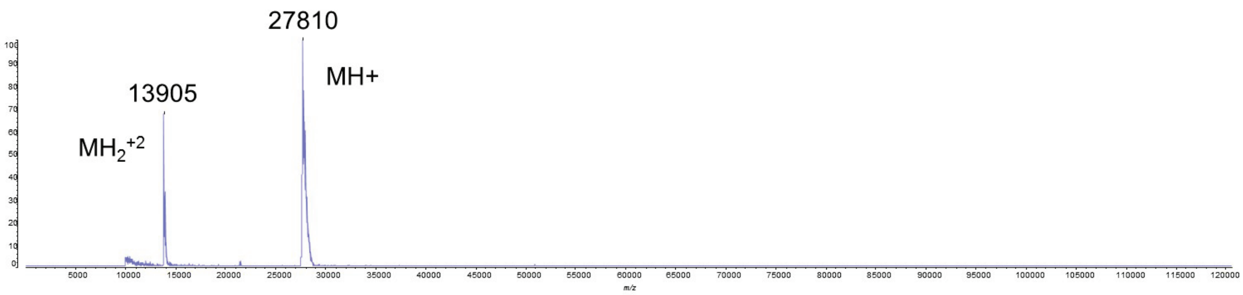

Figure S3. The MALDI-TOF analysis of purified sfGFP-151Phe-K variant. The expected molecular weight is 27860 Da.

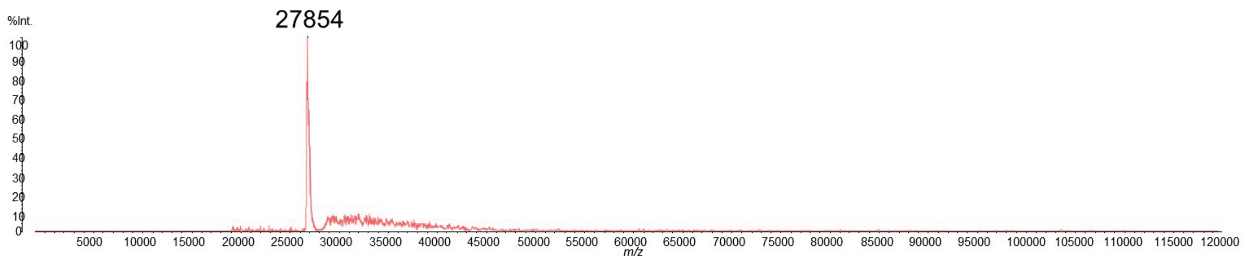

Figure S4. The MALDI-TOF analysis of purified sfGFP-151Met-K variant. The expected molecular weight is 27844 Da.

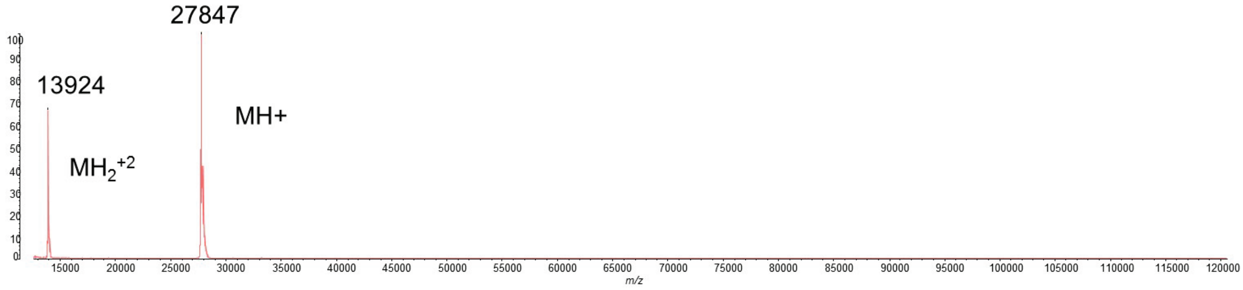

Figure S5. The MALDI-TOF analysis of purified sfGFP-151Pro-K variant. The expected molecular weight is 27810 Da.

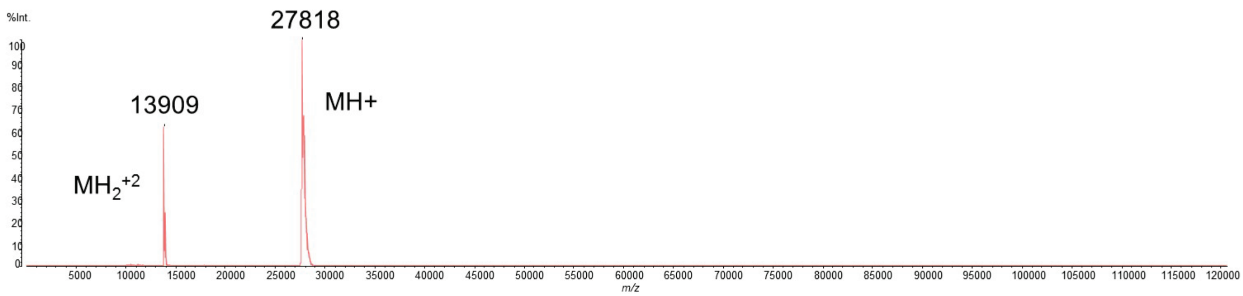

Figure S6. The MALDI-TOF analysis of purified sfGFP-151Ser-K variant. The expected molecular weight is 27800 Da.

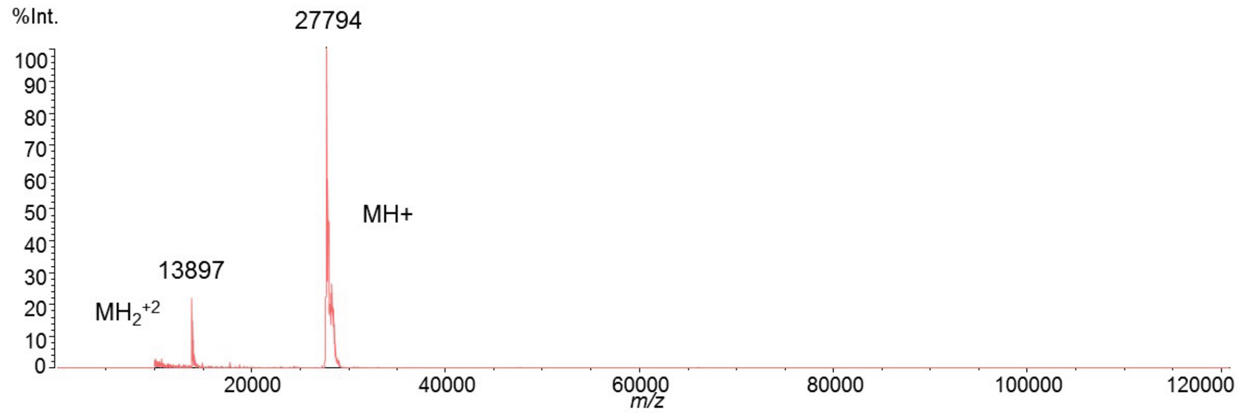

Figure S7. The MALDI-TOF analysis of purified sfGFP-151Thr-K variant. The expected molecular weight is 27814 Da.

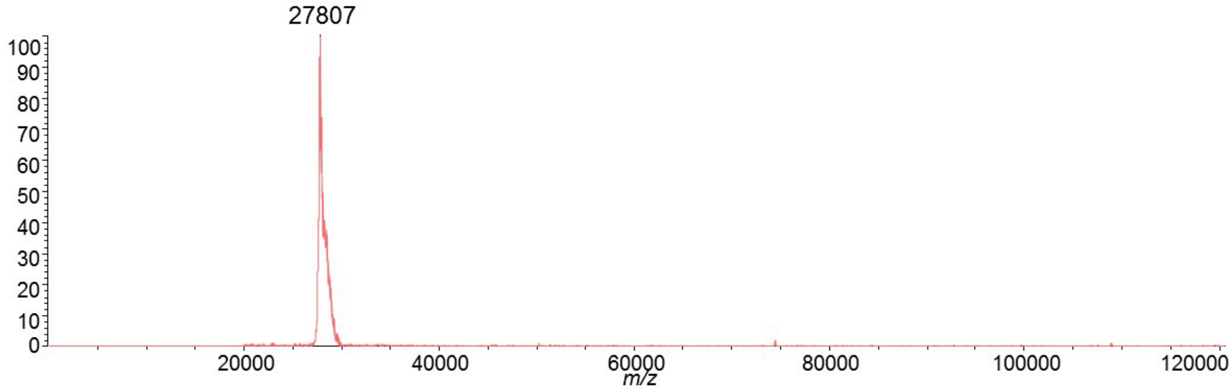

Figure S8. The MALDI-TOF analysis of purified sfGFP-151Trp-K variant. The expected molecular weight is 27899 Da.

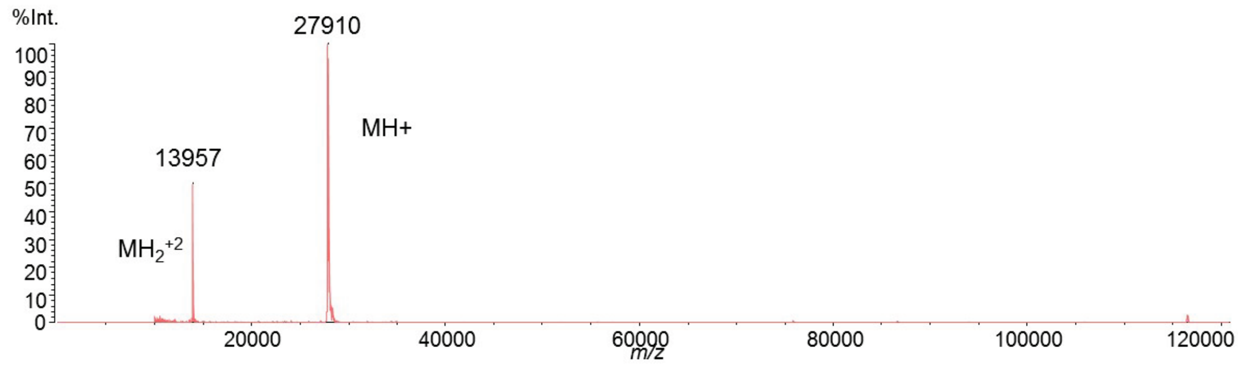

Figure S9. The MALDI-TOF analysis of purified sfGFP-151Tyr-K variant. The expected molecular weight is 27876 Da.

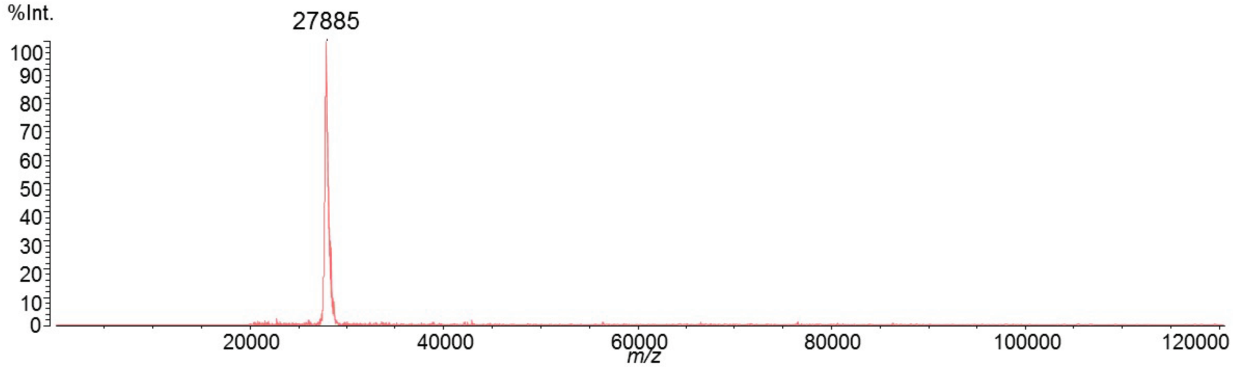

Figure S10. The MALDI-TOF analysis of purified sfGFP-151Val-K variant. The expected molecular weight is 27812 Da.

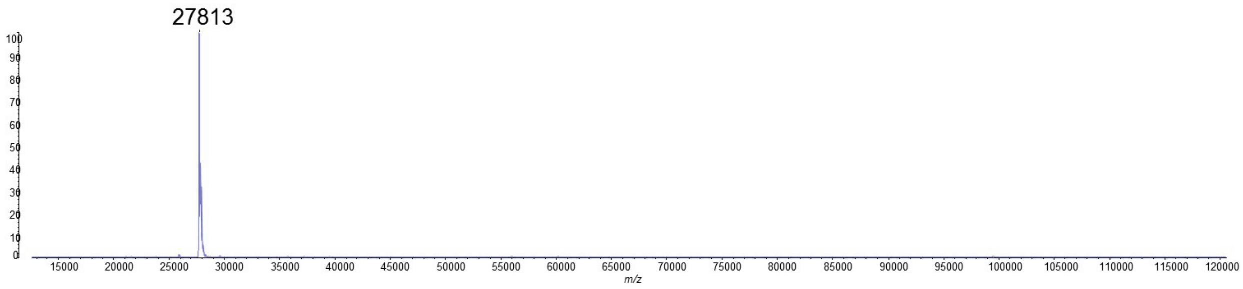

Figure S11. LC-MS/MS analysis of sfGFP-151Ala-K. The tandem mass spectrum of the peptide (residues 141-156) LEYNFNSHNVKITADK from purified sfGFP-151Ala-K. K^{AL} denotes Ala-K incorporation. The partial sequence of the peptide containing the Ala-K can be read from the annotated a, b, or y ion series. Matched peaks are in red.

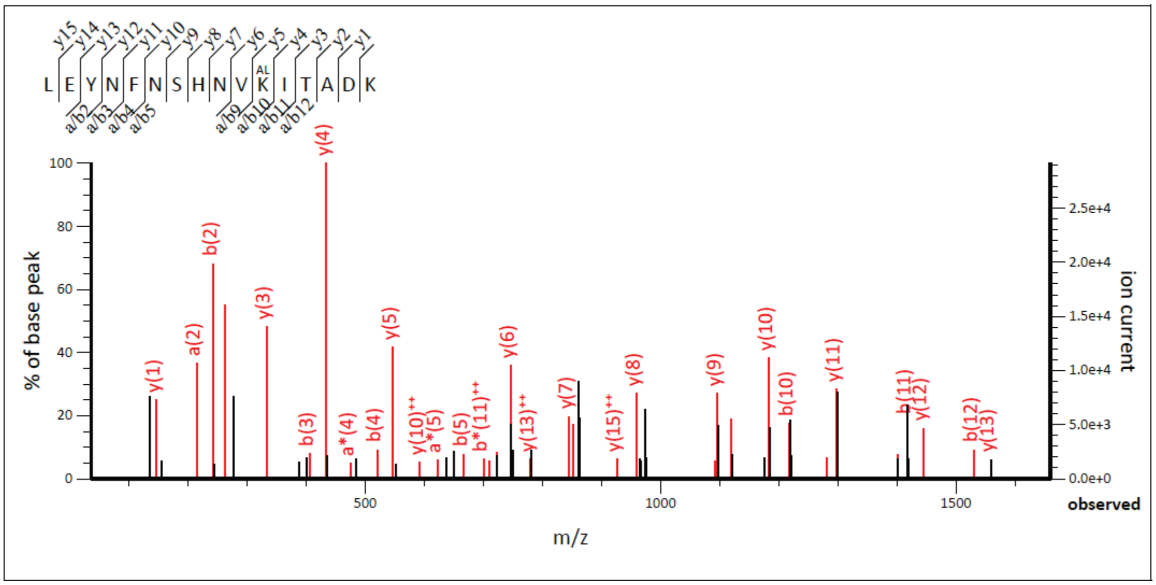

#	a	a^{++}	a*	a^{*++}	b	b^{++}	b*	b^{*++}	Seq.	y	y^{++}	y*	y^{*++}	#
1	86.0964	43.5519			114.0913	57.5493			L					16
2	**215.1390**	108.0731			**243.1339**	122.0706			E	1850.9032	**925.9552**	1833.8766	917.4419	15
3	378.2023	189.6048			**406.1973**	203.6023			Y	1721.8606	**861.4339**	1704.8340	**852.9206**	14
4	492.2453	246.6263	**475.2187**	238.1130	**520.2402**	260.6237	503.2136	252.1105	N	**1558.7972**	**779.9023**	1541.7707	771.3890	13
5	639.3137	320.1605	**622.2871**	311.6472	**667.3086**	334.1579	650.2821	325.6447	F	**1444.7543**	**722.8808**	1427.7278	714.3675	12
6	753.3566	377.1819	736.3301	368.6687	781.3515	391.1794	764.3250	382.6661	N	**1297.6859**	649.3466	**1280.6593**	640.8333	11
7	840.3886	420.6980	823.3621	412.1847	868.3836	434.6954	851.3570	426.1821	S	**1183.6430**	**592.3251**	1166.6164	583.8118	10
8	977.4476	489.2274	960.4210	480.7141	1005.4425	503.2249	988.4159	494.7116	H	**1096.6109**	548.8091	1079.5844	540.2958	9
9	**1091.4905**	546.2489	1074.4639	537.7356	**1119.4854**	560.2463	1102.4588	551.7331	N	**959.5520**	480.2796	942.5255	471.7664	8
10	1190.5589	595.7831	1173.5323	587.2698	**1218.5538**	609.7805	1201.5273	601.2673	V	**845.5091**	423.2582	828.4825	414.7449	7
11	1389.6910	695.3491	1372.6644	686.8359	**1417.6859**	**709.3466**	**1400.6593**	**700.8333**	K	**746.4407**	373.7240	729.4141	365.2107	6
12	1502.7750	751.8912	1485.7485	743.3779	**1530.7700**	765.8886	1513.7434	757.3753	I	**547.3086**	274.1579	530.2821	265.6447	5
13	1603.8227	802.4150	1586.7962	793.9017	1631.8176	816.4125	1614.7911	807.8992	T	**434.2245**	217.6159	417.1980	209.1026	4
14	1674.8598	837.9336	1657.8333	829.4203	1702.8547	851.9310	1685.8282	843.4177	A	**333.1769**	167.0921	316.1503	158.5788	3
15	1789.8868	895.4470	1772.8602	886.9338	1817.8817	909.4445	1800.8551	900.9312	D	**262.1397**	131.5735	245.1132	123.0602	2
16									K	**147.1128**	74.0600	130.0863	65.5468	1

Figure S12. LC-MS/MS analysis of sfGFP-151Cys-K. The tandem mass spectrum of the peptide (residues 141-156) LEYNFNSHNVKITADK from purified sfGFP-151Cys-K. K^{CY} denotes Cys-K incorporation. The partial sequence of the peptide containing the Cys-K can be read from the annotated a, b, or y ion series. Matched peaks are in red.

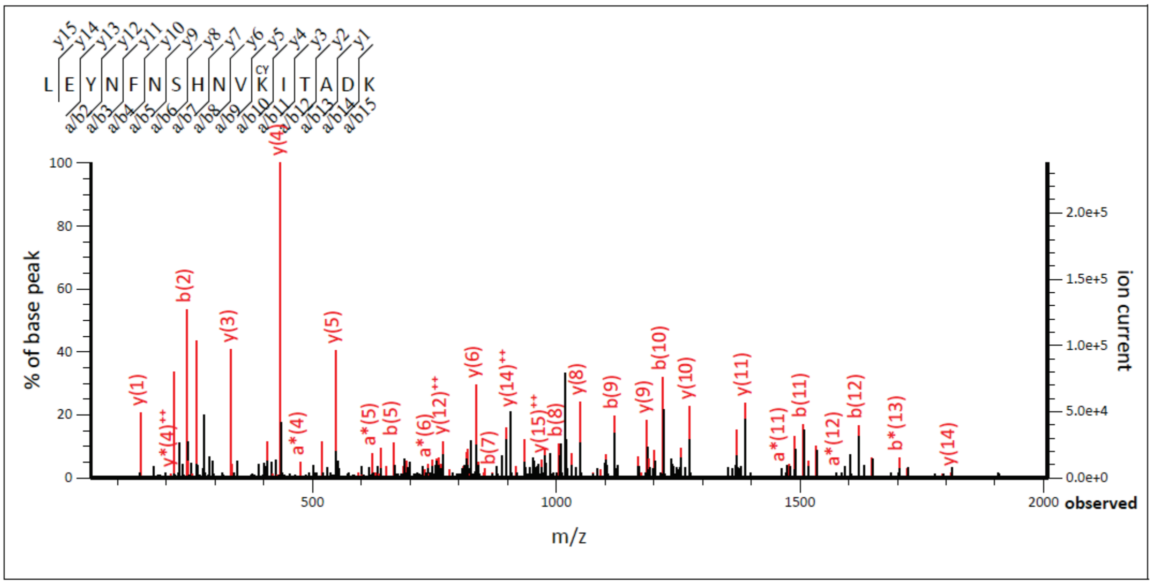

#	a	a++	a*	a*++	b	b++	b*	b*++	Seq.	y	y++	y*	y*++	#
1	86.0964	43.5519			114.0913	57.5493			L					16
2	215.1390	108.0731			243.1339	122.0706			E	1939.8967	970.4520	1922.8701	961.9387	15
3	378.2023	189.6048			406.1973	203.6023			Y	1810.8541	905.9307	1793.8275	897.4174	14
4	492.2453	246.6263	475.2187	238.1130	520.2402	260.6237	503.2136	252.1105	N	1647.7908	824.3990	1630.7642	815.8857	13
5	639.3137	320.1605	622.2871	311.6472	667.3086	334.1579	650.2821	325.6447	F	1533.7478	767.3776	1516.7213	758.8643	12
6	753.3566	377.1819	736.3301	368.6687	781.3515	391.1794	764.3250	382.6661	N	1386.6794	693.8433	1369.6529	685.3301	11
7	840.3886	420.6980	823.3621	412.1847	868.3836	434.6954	851.3570	426.1821	S	1272.6365	636.8219	1255.6099	628.3086	10
8	977.4476	489.2274	960.4210	480.7141	1005.4425	503.2249	988.4159	494.7116	H	1185.6045	593.3059	1168.5779	584.7926	9
9	1091.4905	546.2489	1074.4639	537.7356	1119.4854	560.2463	1102.4588	551.7331	N	1048.5456	524.7764	1031.5190	516.2631	8
10	1190.5589	595.7831	1173.5323	587.2698	1218.5538	609.7805	1201.5273	601.2673	V	934.5026	467.7550	917.4761	459.2417	7
11	1478.6845	739.8459	1461.6580	731.3326	1506.6794	753.8433	1489.6529	745.3301	K	835.4342	418.2207	818.4077	409.7075	6
12	1591.7686	796.3879	1574.7420	787.8746	1619.7635	810.3854	1602.7369	801.8721	I	547.3086	274.1579	530.2821	265.6447	5
13	1692.8163	846.9118	1675.7897	838.3985	1720.8112	860.9092	1703.7846	852.3959	T	434.2245	217.6159	417.1980	209.1026	4
14	1763.8534	882.4303	1746.8268	873.9170	1791.8483	896.4278	1774.8217	887.9145	A	333.1769	167.0921	316.1503	158.5788	3
15	1878.8803	939.9438	1861.8538	931.4305	1906.8752	953.9412	1889.8487	945.4280	D	262.1397	131.5735	245.1132	123.0602	2
16									K	147.1128	74.0600	130.0863	65.5468	1

Figure S13. LC-MS/MS analysis of sfGFP-151Phe-K. The tandem mass spectrum of the peptide (residues 141-156) LEYNFNSHNVKITADK from purified sfGFP-151Phe-K. K^{PH} denotes Phe-K incorporation. The partial sequence of the peptide containing the Phe-K can be read from the annotated a, b, or y ion series. Matched peaks are in red.

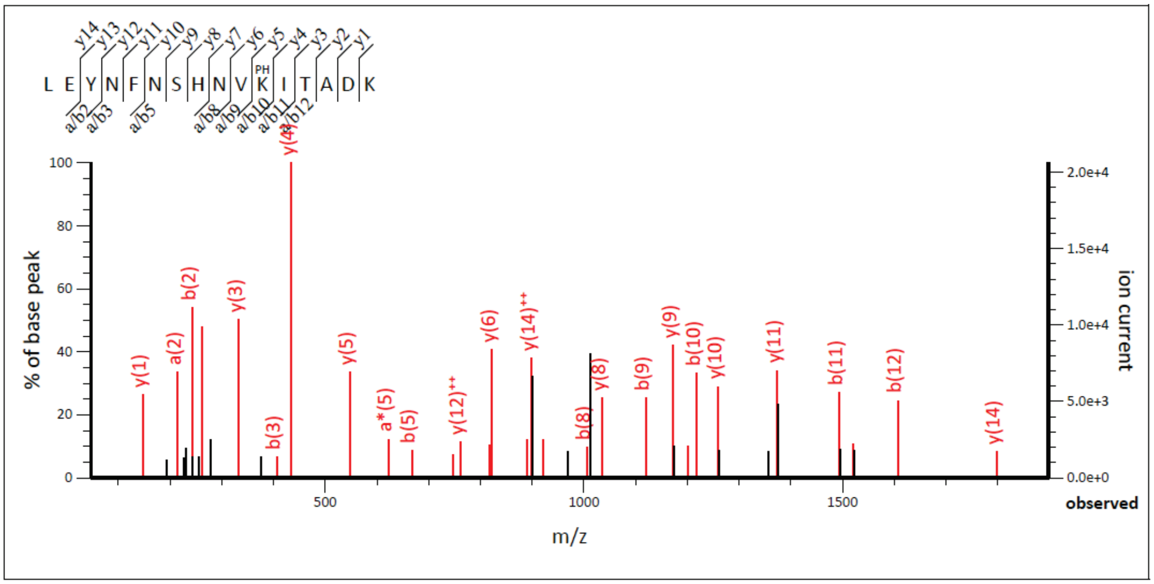

#	a	a++	a*	a*++	b	b++	b*	b*++	Seq.	y	y++	y*	y*++	#
1	86.0964	43.5519			114.0913	57.5493			L					16
2	215.1390	108.0731			243.1339	122.0706			E	1926.9345	963.9709	1909.9079	955.4576	15
3	378.2023	189.6048			406.1973	203.6023			Y	1797.8919	899.4496	1780.8653	890.9363	14
4	492.2453	246.6263	475.2187	238.1130	520.2402	260.6237	503.2136	252.1105	N	1634.8285	817.9179	1617.8020	809.4046	13
5	639.3137	320.1605	622.2871	311.6472	667.3086	334.1579	650.2821	325.6447	F	1520.7856	760.8964	1503.7591	752.3832	12
6	753.3566	377.1819	736.3301	368.6687	781.3515	391.1794	764.3250	382.6661	N	1373.7172	687.3622	1356.6906	678.8490	11
7	840.3886	420.6980	823.3621	412.1847	868.3836	434.6954	851.3570	426.1821	S	1259.6743	630.3408	1242.6477	621.8275	10
8	977.4476	489.2274	960.4210	480.7141	1005.4425	503.2249	988.4159	494.7116	H	1172.6422	586.8248	1155.6157	578.3115	9
9	1091.4905	546.2489	1074.4639	537.7356	1119.4854	560.2463	1102.4588	551.7331	N	1035.5833	518.2953	1018.5568	509.7820	8
10	1190.5589	595.7831	1173.5323	587.2698	1218.5538	609.7805	1201.5273	601.2673	V	921.5404	461.2738	904.5138	452.7606	7
11	1465.7223	733.3648	1448.6957	724.8515	1493.7172	747.3622	1476.6906	738.8490	K	822.4720	411.7396	805.4454	403.2264	6
12	1578.8063	789.9068	1561.7798	781.3935	1606.8013	803.9043	1589.7747	795.3910	I	547.3086	274.1579	530.2821	265.6447	5
13	1679.8540	840.4306	1662.8275	831.9174	1707.8489	854.4281	1690.8224	845.9148	T	434.2245	217.6159	417.1980	209.1026	4
14	1750.8911	875.9492	1733.8646	867.4359	1778.8860	889.9467	1761.8595	881.4334	A	333.1769	167.0921	316.1503	158.5788	3
15	1865.9181	933.4627	1848.8915	924.9494	1893.9130	947.4601	1876.8864	938.9469	D	262.1397	131.5735	245.1132	123.0602	2
16									K	147.1128	74.0600	130.0863	65.5468	1

Figure S14. LC-MS/MS analysis of sfGFP-151Met-K. The tandem mass spectrum of the peptide (residues 141-156) LEYNFNSHNVKITADK from purified sfGFP-151Met-K. K^{ME} denotes Met-K incorporation. The partial sequence of the peptide containing the Met-K can be read from the annotated a, b, or y ion series. Matched peaks are in red.

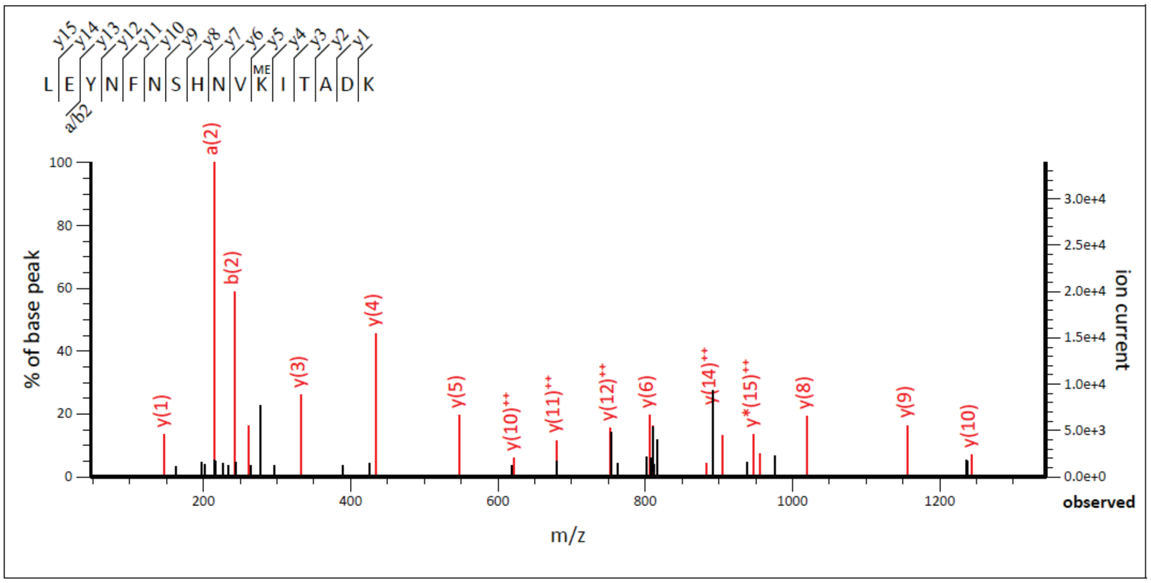

#	a	a++	a*	a*++	b	b++	b*	b*++	Seq.	y	y++	y*	y*++	#
1	86.0964	43.5519			114.0913	57.5493			L					16
2	**215.1390**	108.0731			**243.1339**	122.0706			E	1910.9065	**955.9569**	1893.8800	**947.4436**	15
3	378.2023	189.6048			406.1973	203.6023			Y	1781.8639	**891.4356**	1764.8374	**882.9223**	14
4	492.2453	246.6263	475.2187	238.1130	520.2402	260.6237	503.2136	252.1105	N	1618.8006	**809.9039**	1601.7741	801.3907	13
5	639.3137	320.1605	622.2871	311.6472	667.3086	334.1579	650.2821	325.6447	F	1504.7577	**752.8825**	1487.7311	744.3692	12
6	753.3566	377.1819	736.3301	368.6687	781.3515	391.1794	764.3250	382.6661	N	1357.6893	**679.3483**	1340.6627	670.8350	11
7	840.3886	420.6980	823.3621	412.1847	868.3836	434.6954	851.3570	426.1821	S	**1243.6463**	**622.3268**	1226.6198	613.8135	10
8	977.4476	489.2274	960.4210	480.7141	1005.4425	503.2249	988.4159	494.7116	H	**1156.6143**	578.8108	1139.5878	570.2975	9
9	1091.4905	546.2489	1074.4639	537.7356	1119.4854	560.2463	1102.4588	551.7331	N	**1019.5554**	510.2813	1002.5288	501.7681	8
10	1190.5589	595.7831	1173.5323	587.2698	1218.5538	609.7805	1201.5273	601.2673	V	**905.5125**	453.2599	888.4859	444.7466	7
11	1449.6943	725.3508	1432.6678	716.8375	1477.6893	739.3483	1460.6627	730.8350	K	**806.4441**	403.7257	789.4175	395.2124	6
12	1562.7784	781.8928	1545.7519	773.3796	1590.7733	795.8903	1573.7468	787.3770	I	**547.3086**	274.1579	530.2821	265.6447	5
13	1663.8261	832.4167	1646.7995	823.9034	1691.8210	846.4141	1674.7945	837.9009	T	**434.2245**	217.6159	417.1980	209.1026	4
14	1734.8632	867.9352	1717.8367	859.4220	1762.8581	881.9327	1745.8316	873.4194	A	**333.1769**	167.0921	316.1503	158.5788	3
15	1849.8901	925.4487	1832.8636	916.9354	1877.8851	939.4462	1860.8585	930.9329	D	**262.1397**	131.5735	245.1132	123.0602	2
16									K	**147.1128**	74.0600	130.0863	65.5468	1

Figure S15. LC-MS/MS analysis of sfGFP-151Pro-K. The tandem mass spectrum of the peptide (residues 141-156) LEYNFNSHNVKITADK from purified sfGFP-151Pro-K. K^{PR} denotes Pro-K incorporation. The partial sequence of the peptide containing the Pro-K can be read from the annotated a, b, or y ion series. Matched peaks are in red.

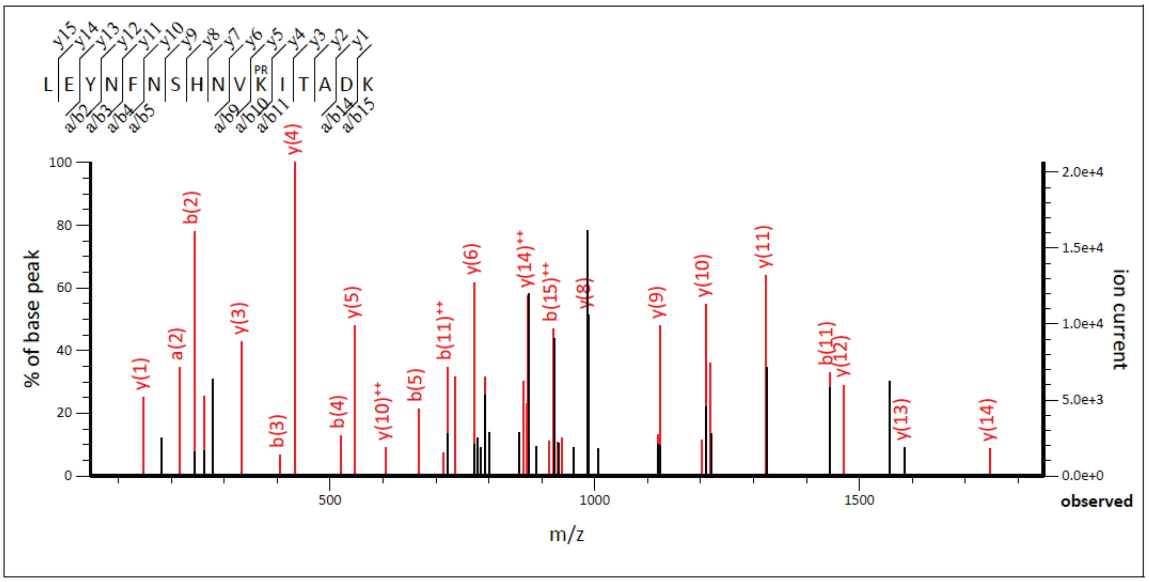

#	a	a++	a*	a*++	b	b++	b*	b*++	Seq.	y	y++	y*	y*++	#
1	86.0964	43.5519			114.0913	57.5493			L					16
2	215.1390	108.0731			243.1339	122.0706			E	1876.9188	938.9630	1859.8923	930.4498	15
3	378.2023	189.6048			406.1973	203.6023			Y	1747.8762	874.4417	1730.8497	865.9285	14
4	492.2453	246.6263	475.2187	238.1130	520.2402	260.6237	503.2136	252.1105	N	1584.8129	792.9101	1567.7863	784.3968	13
5	639.3137	320.1605	622.2871	311.6472	667.3086	334.1579	650.2821	325.6447	F	1470.7700	735.8886	1453.7434	727.3753	12
6	753.3566	377.1819	736.3301	368.6687	781.3515	391.1794	764.3250	382.6661	N	1323.7015	662.3544	1306.6750	653.8411	11
7	840.3886	420.6980	823.3621	412.1847	868.3836	434.6954	851.3570	426.1821	S	1209.6586	605.3329	1192.6321	596.8197	10
8	977.4476	489.2274	960.4210	480.7141	1005.4425	503.2249	988.4159	494.7116	H	1122.6266	561.8169	1105.6000	553.3037	9
9	1091.4905	546.2489	1074.4639	537.7356	1119.4854	560.2463	1102.4588	551.7331	N	985.5677	493.2875	968.5411	484.7742	8
10	1190.5589	595.7831	1173.5323	587.2698	1218.5538	609.7805	1201.5273	601.2673	V	871.5247	436.2660	854.4982	427.7527	7
11	1415.7066	708.3569	1398.6801	699.8437	1443.7015	722.3544	1426.6750	713.8411	K	772.4563	386.7318	755.4298	378.2185	6
12	1528.7907	764.8990	1511.7641	756.3857	1556.7856	778.8964	1539.7591	770.3832	I	547.3086	274.1579	530.2821	265.6447	5
13	1629.8384	815.4228	1612.8118	806.9095	1657.8333	829.4203	1640.8067	820.9070	T	434.2245	217.6159	417.1980	209.1026	4
14	1700.8755	850.9414	1683.8489	842.4281	1728.8704	864.9388	1711.8438	856.4256	A	333.1769	167.0921	316.1503	158.5788	3
15	1815.9024	908.4548	1798.8759	899.9416	1843.8973	922.4523	1826.8708	913.9390	D	262.1397	131.5735	245.1132	123.0602	2
16									K	147.1128	74.0600	130.0863	65.5468	1

Figure S16. LC-MS/MS analysis of sfGFP-151Ser-K. The tandem mass spectrum of the peptide (residues 141-156) LEYNFNSHNVKITADK from purified sfGFP-151Ser-K. K^{SE} denotes Ser-K incorporation. The partial sequence of the peptide containing the Ser-K can be read from the annotated a, b, or y ion series. Matched peaks are in red.

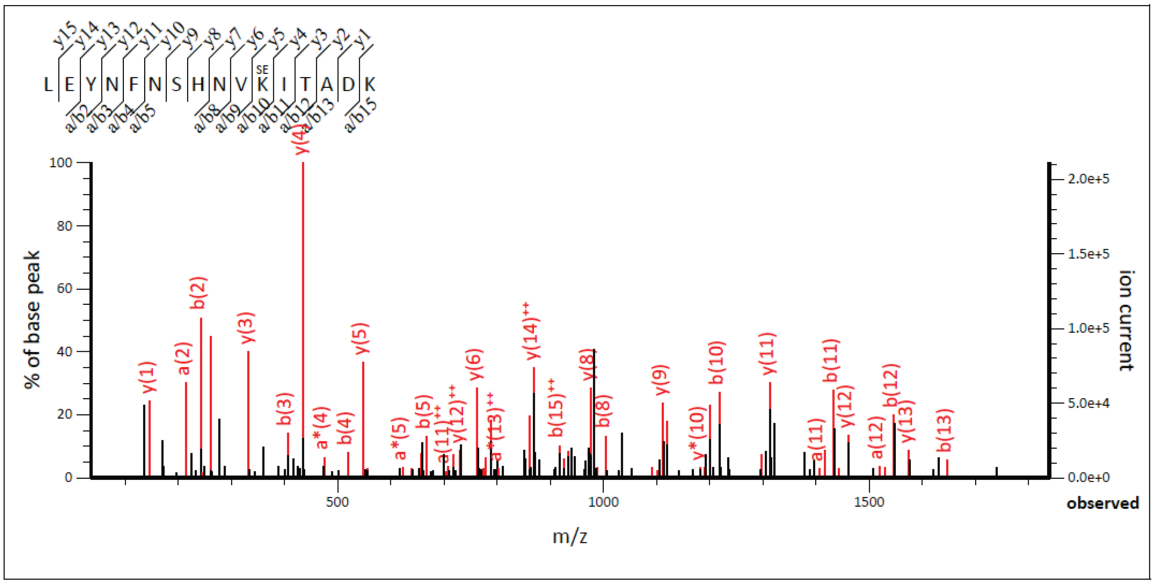

#	a	a++	a*	a*++	b	b++	b*	b*++	Seq.	y	y++	y*	y*++	#
1	86.0964	43.5519			114.0913	57.5493			L					16
2	**215.1390**	108.0731			**243.1339**	122.0706			E	1866.8981	**933.9527**	1849.8715	**925.4394**	15
3	378.2023	189.6048			**406.1973**	203.6023			Y	1737.8555	**869.4314**	1720.8289	**860.9181**	14
4	492.2453	246.6263	**475.2187**	238.1130	**520.2402**	260.6237	503.2136	252.1105	N	**1574.7921**	**787.8997**	1557.7656	**779.3864**	13
5	**639.3137**	320.1605	**622.2871**	311.6472	**667.3086**	334.1579	650.2821	325.6447	F	**1460.7492**	**730.8782**	**1443.7227**	722.3650	12
6	753.3566	377.1819	736.3301	368.6687	781.3515	391.1794	764.3250	382.6661	N	**1313.6808**	**657.3440**	**1296.6543**	648.8308	11
7	840.3886	420.6980	823.3621	412.1847	868.3836	434.6954	851.3570	426.1821	S	**1199.6379**	600.3226	**1182.6113**	591.8093	10
8	**977.4476**	489.2274	960.4210	480.7141	**1005.4425**	503.2249	**988.4159**	494.7116	H	**1112.6058**	**556.8066**	1095.5793	**548.2933**	9
9	**1091.4905**	546.2489	1074.4639	537.7356	**1119.4854**	560.2463	**1102.4588**	551.7331	N	**975.5469**	488.2771	958.5204	479.7638	8
10	**1190.5589**	595.7831	1173.5323	587.2698	**1218.5538**	609.7805	1201.5273	601.2673	V	**861.5040**	431.2556	844.4775	422.7424	7
11	**1405.6859**	**703.3466**	1388.6593	694.8333	**1433.6808**	**717.3440**	**1416.6543**	**708.8308**	K	**762.4356**	381.7214	745.4090	373.2082	6
12	**1518.7700**	759.8886	1501.7434	751.3753	**1546.7649**	**773.8861**	**1529.7383**	765.3728	I	**547.3086**	274.1579	530.2821	265.6447	5
13	1619.8176	810.4125	1602.7911	**801.8992**	**1647.8125**	824.4099	1630.7860	815.8966	T	**434.2245**	217.6159	417.1980	209.1026	4
14	1690.8547	845.9310	1673.8282	837.4177	1718.8497	859.9285	1701.8231	851.4152	A	**333.1769**	167.0921	316.1503	158.5788	3
15	1805.8817	903.4445	1788.8551	894.9312	1833.8766	**917.4419**	1816.8501	908.9287	D	**262.1397**	131.5735	**245.1132**	123.0602	2
16									K	**147.1128**	74.0600	130.0863	65.5468	1

Figure S17. LC-MS/MS analysis of sfGFP-151Thr-K. The tandem mass spectrum of the peptide (residues 141-156) LEYNFNSHNVKITADK from purified sfGFP-151Thr-K. K^{TH} denotes Thr-K incorporation. The partial sequence of the peptide containing the Thr-K can be read from the annotated a, b, or y ion series. Matched peaks are in red.

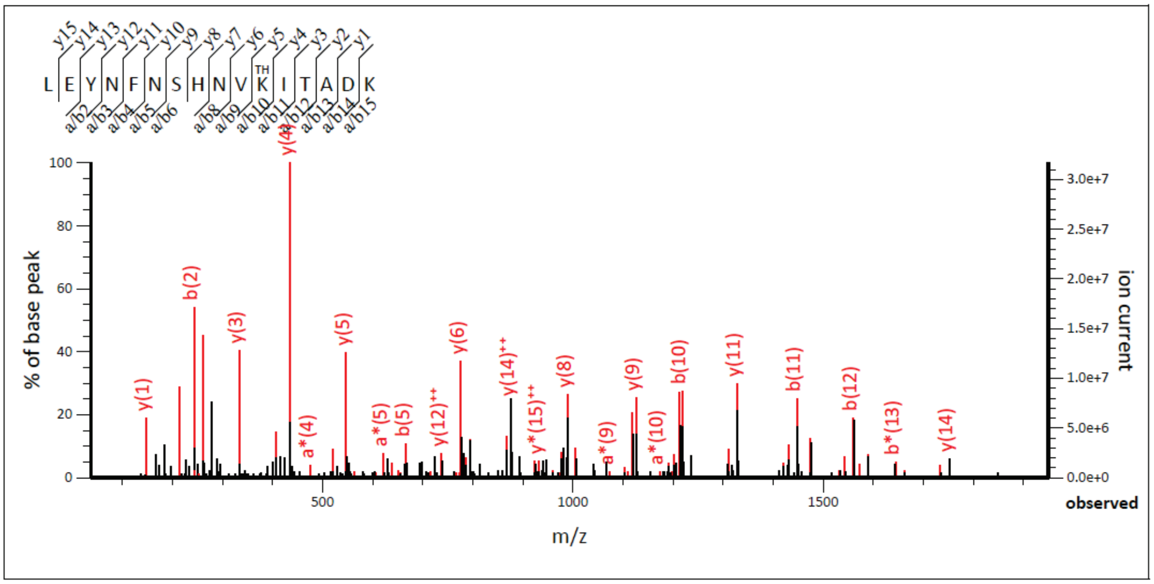

#	a	a++	a*	a*++	b	b++	b*	b*++	Seq.	y	y++	y*	y*++	#
1	86.0964	43.5519			114.0913	57.5493			L					16
2	**215.1390**	108.0731			**243.1339**	122.0706			E	1880.9137	**940.9605**	1863.8872	**932.4472**	15
3	378.2023	189.6048			**406.1973**	203.6023			Y	**1751.8711**	**876.4392**	**1734.8446**	**867.9259**	14
4	**492.2453**	246.6263	**475.2187**	238.1130	**520.2402**	260.6237	**503.2136**	**252.1105**	N	**1588.8078**	**794.9075**	**1571.7812**	**786.3943**	13
5	**639.3137**	320.1605	**622.2871**	311.6472	**667.3086**	334.1579	**650.2821**	325.6447	F	**1474.7649**	**737.8861**	1457.7383	729.3728	12
6	753.3566	377.1819	**736.3301**	368.6687	781.3515	391.1794	764.3250	382.6661	N	**1327.6965**	**664.3519**	**1310.6699**	**655.8386**	11
7	840.3886	420.6980	823.3621	412.1847	868.3836	434.6954	851.3570	426.1821	S	**1213.6535**	**607.3304**	**1196.6270**	598.8171	10
8	**977.4476**	489.2274	**960.4210**	480.7141	**1005.4425**	**503.2249**	988.4159	494.7116	H	**1126.6215**	**563.8144**	**1109.5949**	555.3011	9
9	1091.4905	546.2489	**1074.4639**	537.7356	**1119.4854**	560.2463	**1102.4588**	551.7331	N	**989.5626**	495.2849	**972.5360**	486.7717	8
10	**1190.5589**	595.7831	**1173.5323**	587.2698	**1218.5538**	609.7805	**1201.5273**	601.2673	V	**875.5197**	438.2635	858.4931	429.7502	7
11	**1419.7015**	710.3544	1402.6750	701.8411	**1447.6965**	**724.3519**	**1430.6699**	**715.8386**	K	**776.4512**	388.7293	759.4247	380.2160	6
12	**1532.7856**	**766.8964**	1515.7591	758.3832	**1560.7805**	780.8939	**1543.7540**	**772.3806**	I	**547.3086**	274.1579	530.2821	265.6447	5
13	1633.8333	817.4203	1616.8067	808.9070	**1661.8282**	831.4177	**1644.8016**	822.9045	T	**434.2245**	217.6159	417.1980	209.1026	4
14	1704.8704	852.9388	1687.8438	844.4256	**1732.8653**	**866.9363**	1715.8388	**858.4230**	A	**333.1769**	167.0921	316.1503	158.5788	3
15	1819.8973	910.4523	1802.8708	901.9390	1847.8923	**924.4498**	1830.8657	915.9365	D	**262.1397**	131.5735	**245.1132**	123.0602	2
16									K	**147.1128**	74.0600	130.0863	65.5468	1

Figure S18. LC-MS/MS analysis of sfGFP-151Trp-K. The tandem mass spectrum of the peptide (residues 141-156) LEYNFNSHNVKITADK from purified sfGFP-151Trp-K. K^{TR} denotes Trp-K incorporation. The partial sequence of the peptide containing the Trp-K can be read from the annotated a, b, or y ion series. Matched peaks are in red.

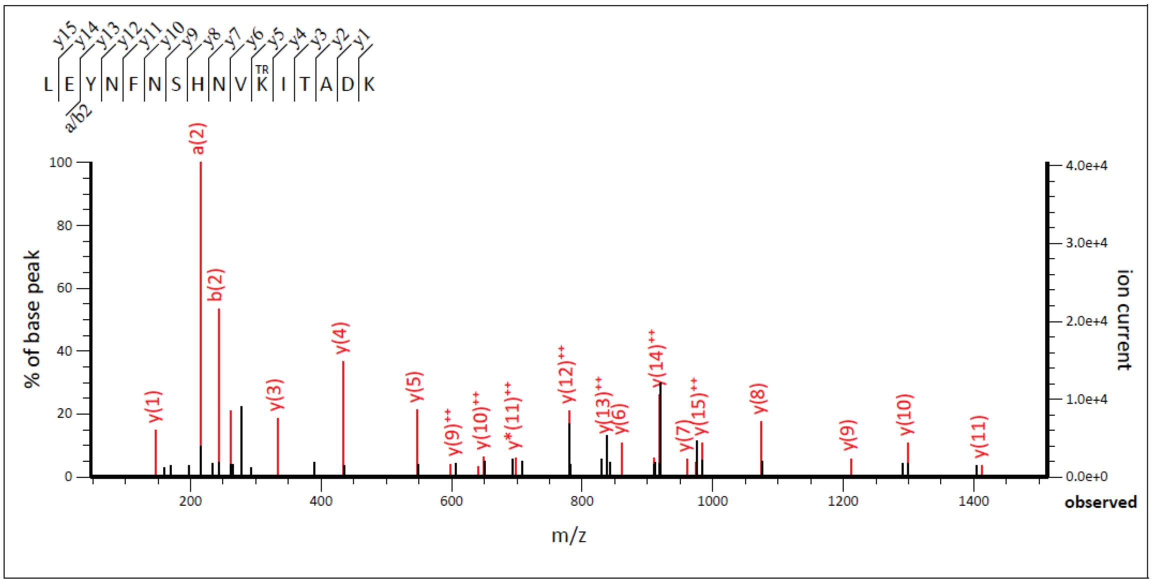

#	a	a^{++}	a^{*}	a^{*++}	b	b^{++}	b^{*}	b^{*++}	Seq.	y	y^{++}	y^{*}	y^{*++}	#
1	86.0964	43.5519			114.0913	57.5493			L					16
2	**215.1390**	108.0731			**243.1339**	122.0706			E	1965.9454	**983.4763**	1948.9188	**974.9630**	15
3	378.2023	189.6048			406.1973	203.6023			Y	1836.9028	**918.9550**	1819.8762	**910.4417**	14
4	492.2453	246.6263	475.2187	238.1130	520.2402	260.6237	503.2136	252.1105	N	1673.8394	**837.4234**	1656.8129	**828.9101**	13
5	639.3137	320.1605	622.2871	311.6472	667.3086	334.1579	650.2821	325.6447	F	1559.7965	**780.4019**	1542.7700	771.8886	12
6	753.3566	377.1819	736.3301	368.6687	781.3515	391.1794	764.3250	382.6661	N	**1412.7281**	706.8677	1395.7015	**698.3544**	11
7	840.3886	420.6980	823.3621	412.1847	868.3836	434.6954	851.3570	426.1821	S	**1298.6852**	**649.8462**	1281.6586	**641.3329**	10
8	977.4476	489.2274	960.4210	480.7141	1005.4425	503.2249	988.4159	494.7116	H	**1211.6531**	**606.3302**	1194.6266	**597.8169**	9
9	1091.4905	546.2489	1074.4639	537.7356	1119.4854	560.2463	1102.4588	551.7331	N	**1074.5942**	537.8007	1057.5677	529.2875	8
10	1190.5589	595.7831	1173.5323	587.2698	1218.5538	609.7805	1201.5273	601.2673	V	**960.5513**	480.7793	943.5247	472.2660	7
11	1504.7332	752.8702	1487.7066	744.3569	1532.7281	766.8677	1515.7015	758.3544	K	**861.4829**	431.2451	844.4563	422.7318	6
12	1617.8172	809.4123	1600.7907	800.8990	1645.8122	823.4097	1628.7856	814.8964	I	**547.3086**	274.1579	530.2821	265.6447	5
13	1718.8649	859.9361	1701.8384	851.4228	1746.8598	873.9336	1729.8333	865.4203	T	**434.2245**	217.6159	417.1980	209.1026	4
14	1789.9020	895.4547	1772.8755	886.9414	1817.8969	909.4521	1800.8704	900.9388	A	**333.1769**	167.0921	316.1503	158.5788	3
15	1904.9290	952.9681	1887.9024	944.4548	1932.9239	966.9656	1915.8973	958.4523	D	**262.1397**	131.5735	245.1132	123.0602	2
16									K	**147.1128**	74.0600	130.0863	65.5468	1

Figure S19. LC-MS/MS analysis of sfGFP-151Tyr-K. The tandem mass spectrum of the peptide (residues 141-156) LEYNFNSHNVKITADK from purified sfGFP-151Tyr-K. K^{TY} denotes Tyr-K incorporation. The partial sequence of the peptide containing the Tyr-K can be read from the annotated a, b, or y ion series. Matched peaks are in red.

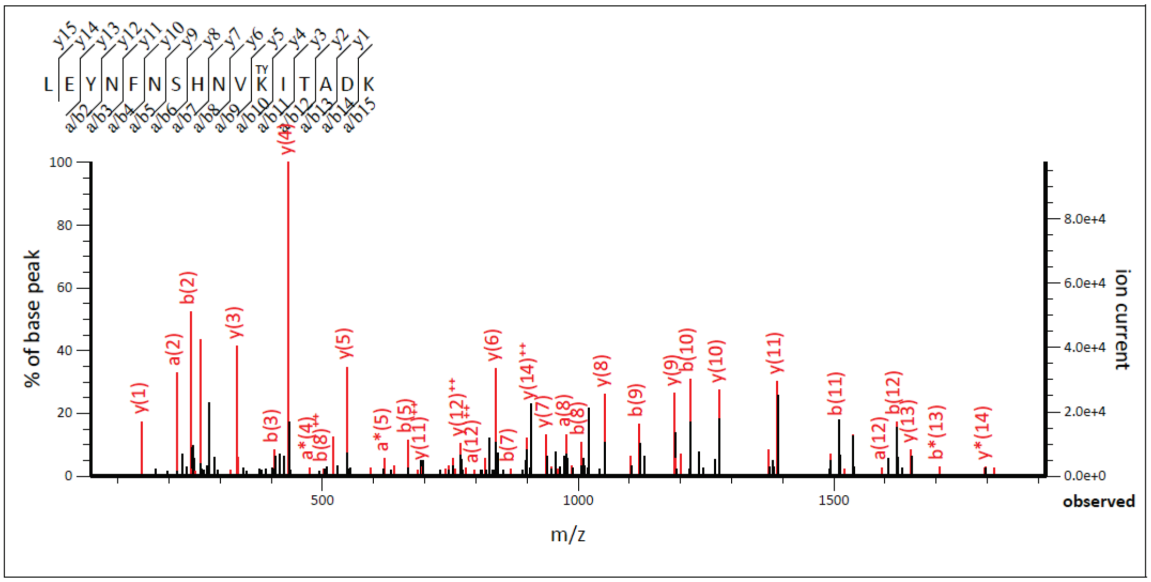

#	a	a^{++}	a*	a*$^{++}$	b	b^{++}	b*	b*$^{++}$	Seq.	y	y^{++}	y*	y*$^{++}$	#
1	86.0964	43.5519			114.0913	57.5493			L					16
2	**215.1390**	108.0731			**243.1339**	122.0706			E	1942.9294	**971.9683**	1925.9028	**963.4550**	15
3	378.2023	189.6048			**406.1973**	203.6023			Y	**1813.8868**	**907.4470**	**1796.8602**	**898.9338**	14
4	492.2453	246.6263	**475.2187**	238.1130	**520.2402**	260.6237	**503.2136**	**252.1105**	N	**1650.8234**	**825.9154**	1633.7969	**817.4021**	13
5	**639.3137**	**320.1605**	**622.2871**	311.6472	**667.3086**	**334.1579**	650.2821	325.6447	F	**1536.7805**	**768.8939**	**1519.7540**	**760.3806**	12
6	753.3566	377.1819	736.3301	368.6687	**781.3515**	391.1794	764.3250	382.6661	N	**1389.7121**	**695.3597**	**1372.6856**	**686.8464**	11
7	840.3886	420.6980	823.3621	412.1847	**868.3836**	434.6954	851.3570	426.1821	S	**1275.6692**	638.3382	1258.6426	629.8250	10
8	**977.4476**	489.2274	**960.4210**	480.7141	**1005.4425**	**503.2249**	**988.4159**	494.7116	H	**1188.6371**	**594.8222**	1171.6106	586.3089	9
9	1091.4905	546.2489	1074.4639	537.7356	**1119.4854**	560.2463	**1102.4588**	551.7331	N	**1051.5782**	526.2928	1034.5517	517.7795	8
10	**1190.5589**	595.7831	1173.5323	587.2698	**1218.5538**	609.7805	**1201.5273**	601.2673	V	**937.5353**	469.2713	920.5088	460.7580	7
11	1481.7172	**741.3622**	1464.6906	732.8490	**1509.7121**	**755.3597**	**1492.6856**	**746.8464**	K	**838.4669**	419.7371	821.4403	411.2238	6
12	**1594.8013**	**797.9043**	1577.7747	789.3910	**1622.7962**	811.9017	**1605.7696**	803.3884	I	**547.3086**	274.1579	530.2821	265.6447	5
13	1695.8489	848.4281	1678.8224	839.9148	1723.8438	862.4256	**1706.8173**	853.9123	T	**434.2245**	217.6159	417.1980	209.1026	4
14	1766.8860	883.9467	1749.8595	875.4334	**1794.8810**	897.9441	1777.8544	889.4308	A	**333.1769**	167.0921	316.1503	158.5788	3
15	1881.9130	941.4601	1864.8864	932.9469	1909.9079	**955.4576**	1892.8814	**946.9443**	D	**262.1397**	131.5735	**245.1132**	123.0602	2
16									K	**147.1128**	74.0600	130.0863	65.5468	1

Figure S20. LC-MS/MS analysis of sfGFP-151Val-K. The tandem mass spectrum of the peptide (residues 141-156) LEYNFNSHNVKITADK from purified sfGFP-151Val-K. K^{VA} denotes Val-K incorporation. The partial sequence of the peptide containing the Val-K can be read from the annotated a, b, or y ion series. Matched peaks are in red.

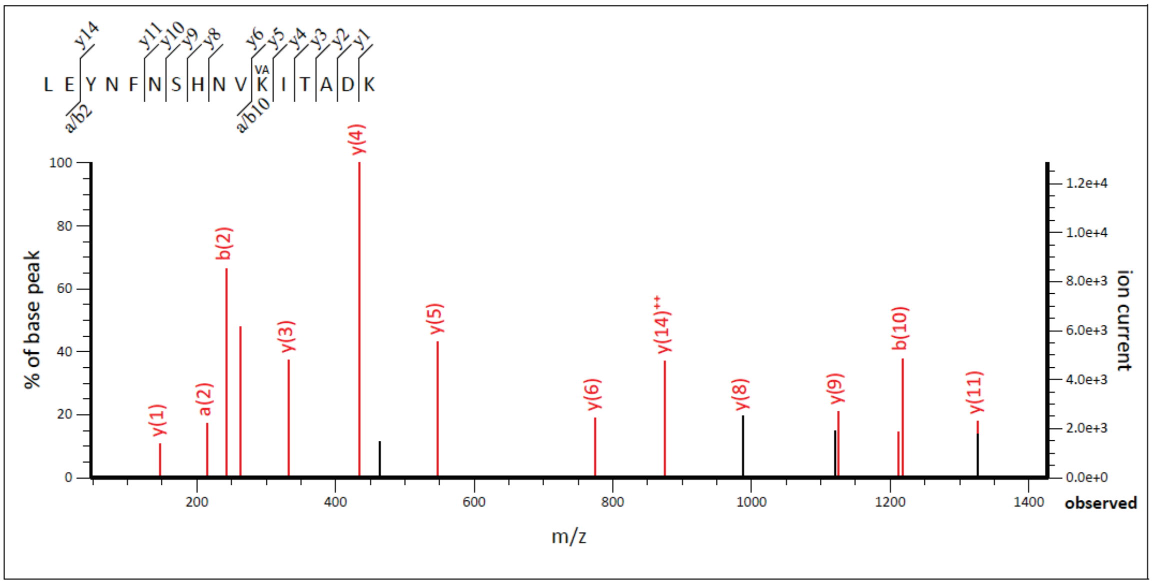

#	a	a++	a*	a*++	b	b++	b*	b*++	Seq.	y	y++	y*	y*++	#
1	86.0964	43.5519			114.0913	57.5493			L					16
2	**215.1390**	108.0731			**243.1339**	122.0706			E	1878.9345	939.9709	1861.9079	931.4576	15
3	378.2023	189.6048			406.1973	203.6023			Y	1749.8919	**875.4496**	1732.8653	866.9363	14
4	492.2453	246.6263	475.2187	238.1130	520.2402	260.6237	503.2136	252.1105	N	1586.8285	793.9179	1569.8020	785.4046	13
5	639.3137	320.1605	622.2871	311.6472	667.3086	334.1579	650.2821	325.6447	F	1472.7856	736.8964	1455.7591	728.3832	12
6	753.3566	377.1819	736.3301	368.6687	781.3515	391.1794	764.3250	382.6661	N	**1325.7172**	663.3622	1308.6906	654.8490	11
7	840.3886	420.6980	823.3621	412.1847	868.3836	434.6954	851.3570	426.1821	S	**1211.6743**	606.3408	1194.6477	597.8275	10
8	977.4476	489.2274	960.4210	480.7141	1005.4425	503.2249	988.4159	494.7116	H	**1124.6422**	562.8248	1107.6157	554.3115	9
9	1091.4905	546.2489	1074.4639	537.7356	1119.4854	560.2463	1102.4588	551.7331	N	**987.5833**	494.2953	970.5568	485.7820	8
10	1190.5589	595.7831	1173.5323	587.2698	**1218.5538**	609.7805	1201.5273	601.2673	V	873.5404	437.2738	856.5138	428.7606	7
11	1417.7223	709.3648	1400.6957	700.8515	1445.7172	723.3622	1428.6906	714.8490	K	**774.4720**	387.7396	757.4454	379.2264	6
12	1530.8063	765.9068	1513.7798	757.3935	1558.8013	779.9043	1541.7747	771.3910	I	**547.3086**	274.1579	530.2821	265.6447	5
13	1631.8540	816.4306	1614.8275	807.9174	1659.8489	830.4281	1642.8224	821.9148	T	**434.2245**	217.6159	417.1980	209.1026	4
14	1702.8911	851.9492	1685.8646	843.4359	1730.8860	865.9467	1713.8595	857.4334	A	**333.1769**	167.0921	316.1503	158.5788	3
15	1817.9181	909.4627	1800.8915	900.9494	1845.9130	923.4601	1828.8864	914.9469	D	**262.1397**	131.5735	245.1132	123.0602	2
16									K	**147.1128**	74.0600	130.0863	65.5468	1

Figure S21. The MALDI-TOF analyses of purified WT PKM2 and its valylated variants. The expected molecular weight difference is 99 Da.

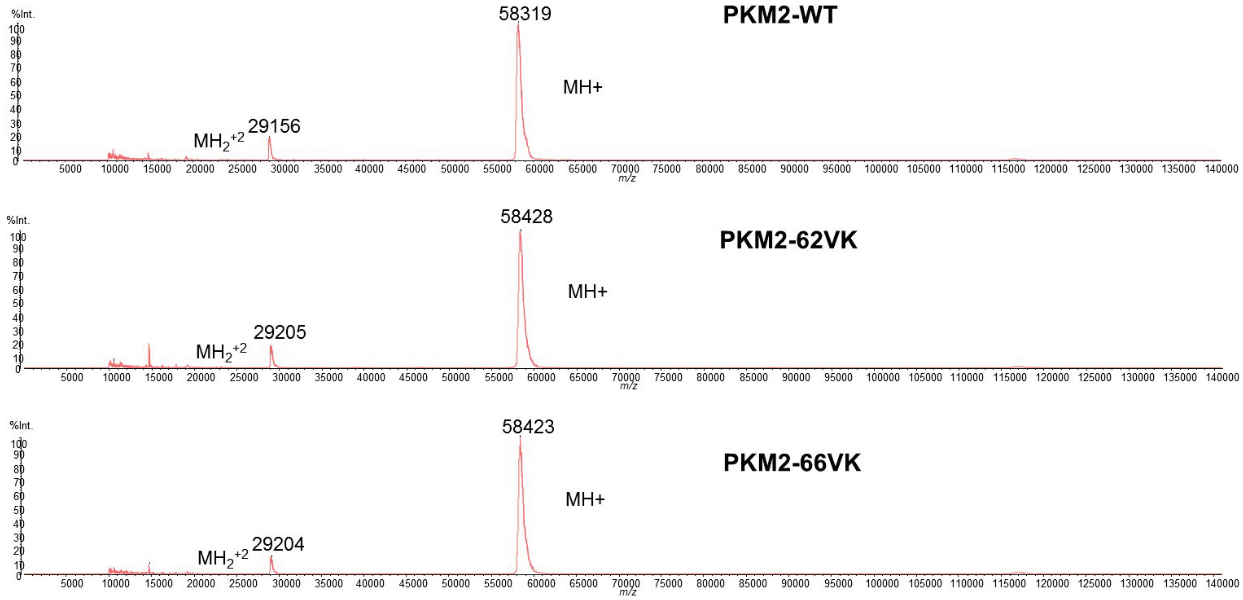

Figure S22. The MALDI-TOF analyses of purified WT G6PD and its tyrosylated variant. The expected molecular weight difference is 163 Da.

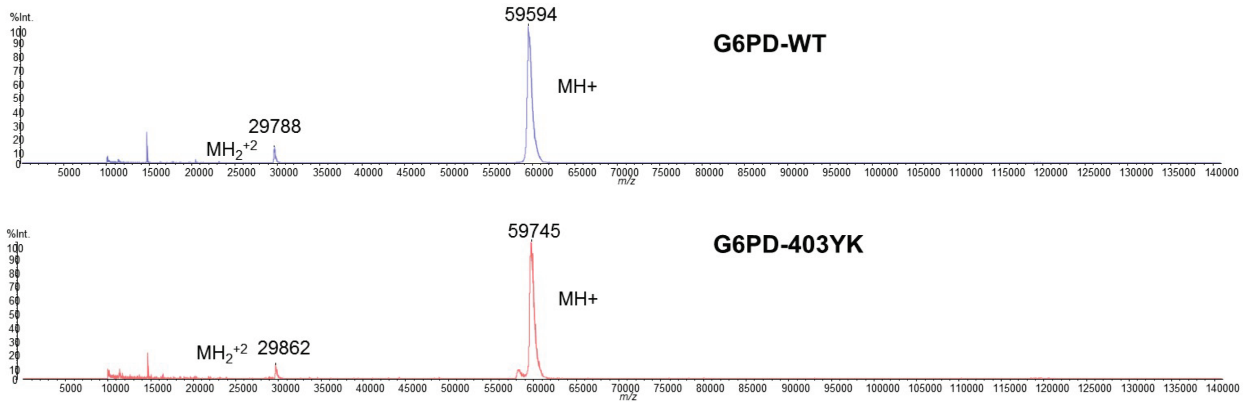

Figure S23. LC-MS/MS analysis of PKM2-62Val-K expressed in BL21(DE3) cells. The tandem mass spectrum of the peptide (residues 57-66) SVETLKEMIK from purified PKM2-62Val-K. K^{VA} denotes Val-K incorporation. M^{OX} denotes oxidation of methionine. The partial sequence of the peptide containing the Val-K can be read from the annotated a, b, or y ion series. Matched peaks are in red.

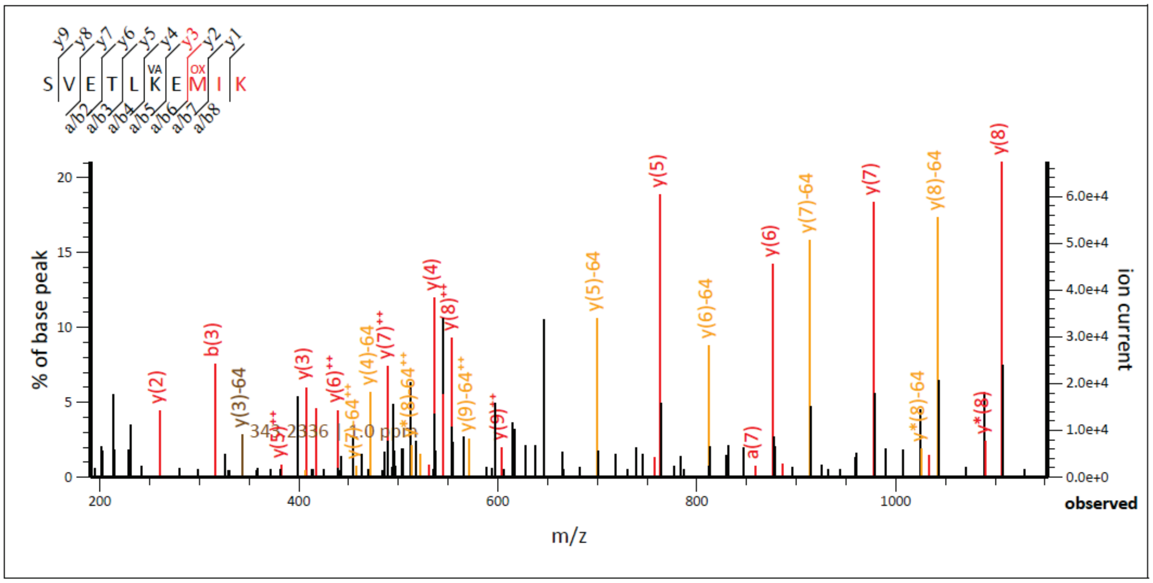

#	a	a^{++}	a*	a^{*++}	b	b^{++}	b*	b^{*++}	Seq.	y	y^{++}	y*	y^{*++}	#
1	60.0444	30.5258			88.0393	44.5233			S					10
2	**159.1128**	80.0600			**187.1077**	94.0575			V	1205.6810	**603.3441**	1188.6544	594.8309	9
3	288.1554	144.5813			**316.1503**	158.5788			E	**1106.6126**	**553.8099**	**1089.5860**	**545.2967**	8
4	389.2031	195.1052			**417.1980**	209.1026			T	**977.5700**	**489.2886**	960.5434	480.7754	7
5	502.2871	251.6472			**530.2821**	265.6447			L	**876.5223**	**438.7648**	859.4958	430.2515	6
6	729.4505	365.2289	712.4240	356.7156	**757.4454**	379.2264	740.4189	370.7131	K	**763.4382**	**382.2228**	746.4117	373.7095	5
7	**858.4931**	429.7502	841.4666	421.2369	**886.4880**	443.7477	869.4615	435.2344	E	**536.2749**	268.6411	519.2483	260.1278	4
8	1005.5285	503.2679	988.5020	494.7546	**1033.5234**	517.2654	1016.4969	508.7521	M	**407.2323**	204.1198	390.2057	195.6065	3
9	1118.6126	559.8099	1101.5860	551.2967	1146.6075	573.8074	1129.5809	565.2941	I	**260.1969**	130.6021	243.1703	122.0888	2
10									K	**147.1128**	74.0600	**130.0863**	65.5468	1

Figure S24. LC-MS/MS analysis of PKM2-66Val-K expressed in BL21(DE3) cells. The tandem mass spectrum of the peptide (residues 63-73) EMIKSGMNVAR from purified PKM2-66Val-K. K^{VA} denotes Val-K incorporation. M^{OX} denotes oxidation of methionine. The partial sequence of the peptide containing the Val-K can be read from the annotated a, b, or y ion series. Matched peaks are in red.

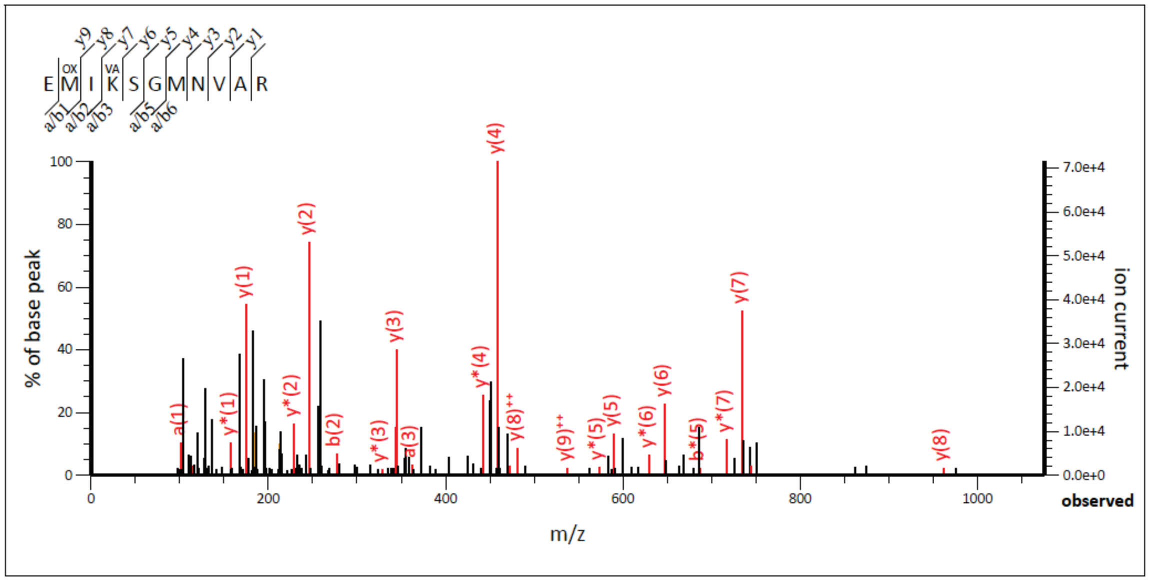

#	a	a^{++}	a*	a^{*++}	b	b^{++}	b*	b^{*++}	Seq.	y	y^{++}	y*	y^{*++}	#
1	**102.0550**	51.5311			130.0499	65.5286			E					11
2	249.0904	125.0488			**277.0853**	139.0463			M	1221.6442	611.3258	1204.6177	602.8125	10
3	**362.1744**	181.5908			390.1693	195.5883			I	1074.6088	**537.8081**	1057.5823	529.2948	9
4	589.3378	295.1725	572.3112	286.6593	617.3327	309.1700	600.3062	300.6567	K	**961.5248**	**481.2660**	944.4982	**472.7527**	8
5	676.3698	338.6885	659.3433	330.1753	704.3647	352.6860	**687.3382**	**344.1727**	S	**734.3614**	367.6843	**717.3348**	359.1711	7
6	733.3913	367.1993	716.3647	358.6860	761.3862	381.1967	**744.3597**	372.6835	G	**647.3294**	324.1683	**630.3028**	315.6550	6
7	864.4318	432.7195	847.4052	424.2062	892.4267	446.7170	875.4001	438.2037	M	**590.3079**	295.6576	**573.2813**	287.1443	5
8	978.4747	489.7410	961.4482	481.2277	1006.4696	503.7384	989.4431	495.2252	N	**459.2674**	**230.1373**	**442.2409**	221.6241	4
9	1077.5431	539.2752	1060.5166	530.7619	1105.5380	553.2727	1088.5115	544.7594	V	**345.2245**	173.1159	**328.1979**	164.6026	3
10	1148.5802	574.7938	1131.5537	566.2805	1176.5751	588.7912	1159.5486	580.2779	A	**246.1561**	123.5817	**229.1295**	**115.0684**	2
11									R	**175.1190**	88.0631	**158.0924**	79.5498	1

Figure S25. LC-MS/MS analysis of G6PD-403Tyr-K expressed in BL21(DE3) cells. The tandem mass spectrum of the peptide (residues 394-407) VQPNEAVYTKMMTK from purified G6PD-403Tyr-K. K^{TY} denotes Tyr-K incorporation. M^{OX} denotes oxidation of methionine. The partial sequence of the peptide containing the Tyr-K can be read from the annotated a, b, or y ion series. Matched peaks are in red.

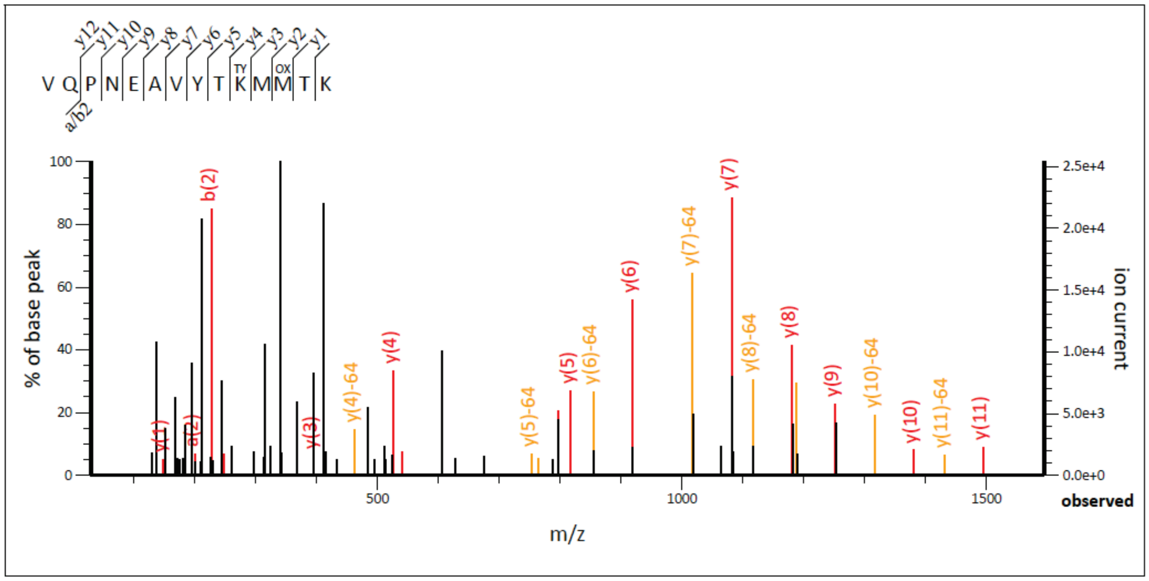

#	a	a^{++}	a*	a*$^{++}$	b	b^{++}	b*	b*$^{++}$	Seq.	y	y^{++}	y*	y*$^{++}$	#
1	72.0808	36.5440			100.0757	50.5415			V					14
2	**200.1394**	100.5733	**183.1128**	92.0600	**228.1343**	114.5708	**211.1077**	106.0575	Q	1719.8081	860.4077	1702.7815	851.8944	13
3	297.1921	149.0997	280.1656	140.5864	325.1870	163.0972	308.1605	154.5839	P	1591.7495	**796.3784**	1574.7229	787.8651	12
4	411.2350	206.1212	394.2085	197.6079	439.2300	220.1186	422.2034	211.6053	N	**1494.6967**	747.8520	1477.6702	739.3387	11
5	540.2776	270.6425	523.2511	262.1292	568.2726	284.6399	551.2460	276.1266	E	**1380.6538**	690.8305	1363.6272	682.3173	10
6	611.3148	306.1610	594.2882	297.6477	639.3097	320.1585	622.2831	311.6452	A	**1251.6112**	626.3092	1234.5846	617.7960	9
7	710.3832	355.6952	693.3566	347.1819	738.3781	369.6927	721.3515	361.1794	V	**1180.5741**	590.7907	1163.5475	582.2774	8
8	873.4465	437.2269	856.4199	428.7136	901.4414	451.2243	884.4149	442.7111	Y	**1081.5057**	**541.2565**	1064.4791	532.7432	7
9	974.4942	487.7507	957.4676	479.2375	1002.4891	501.7482	985.4625	493.2349	T	**918.4423**	459.7248	901.4158	451.2115	6
10	1265.6525	633.3299	1248.6259	624.8166	1293.6474	647.3273	1276.6208	638.8141	K	**817.3947**	409.2010	800.3681	400.6877	5
11	1396.6930	698.8501	1379.6664	690.3368	1424.6879	712.8476	1407.6613	704.3343	M	**526.2364**	263.6218	509.2098	255.1085	4
12	1543.7284	772.3678	1526.7018	763.8545	1571.7233	786.3653	1554.6967	777.8520	M	**395.1959**	198.1016	378.1693	189.5883	3
13	1644.7760	822.8917	1627.7495	814.3784	1672.7709	836.8891	1655.7444	828.3758	T	**248.1605**	124.5839	231.1339	116.0706	2
14									K	**147.1128**	74.0600	130.0863	65.5468	1

Figure S26. The enzyme activity of purified PKM2 and its variants with near-cognate amino acids. 100 ng of purified enzyme was used in each assay. The values of mean and standard deviation were calculated based on three replicates.

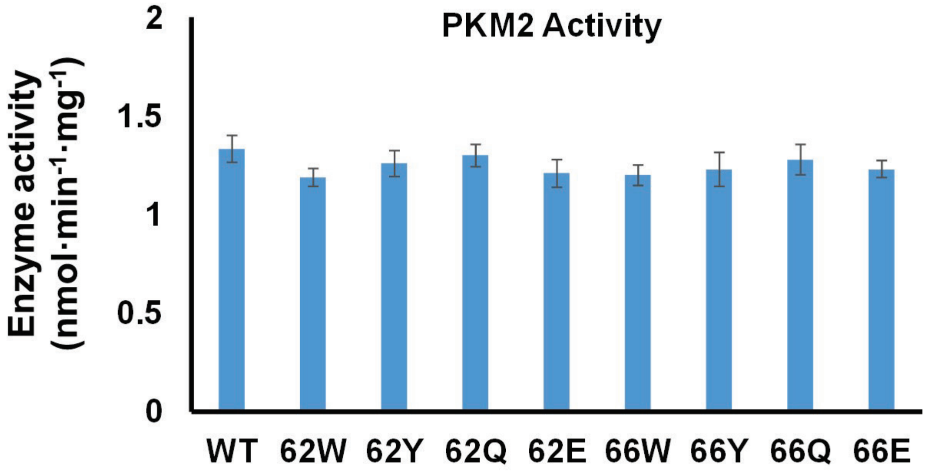

Figure 27. CD spectra of PKM2 and its variants. Scanning was performed from 190 nm to 250 nm with a 60 nm/min speed five times for each sample and the average was plotted.

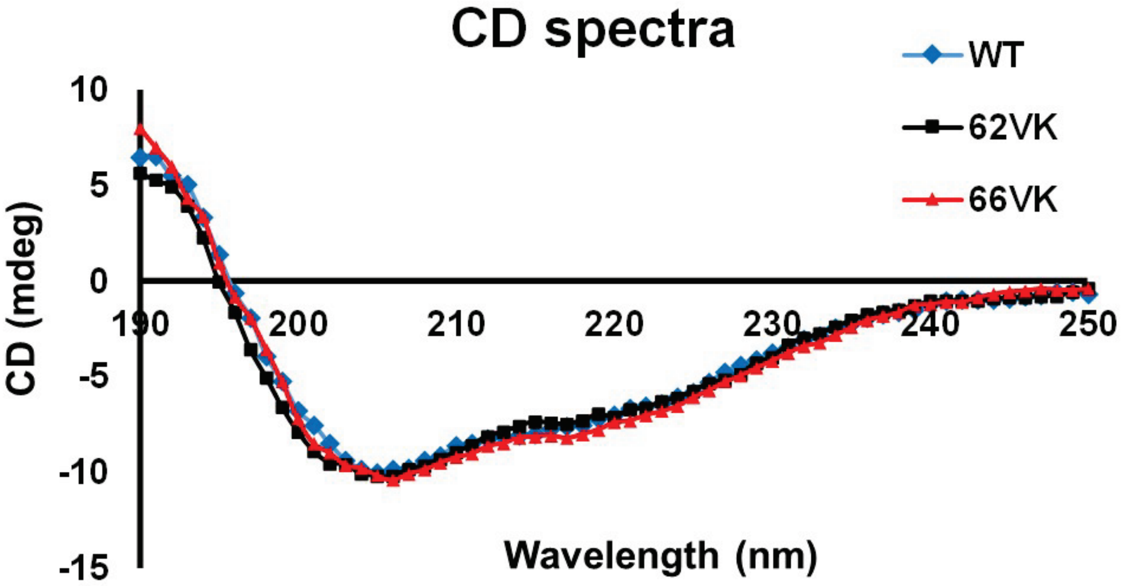

Figure S28. The enzyme activity of purified G6PD and its variants with near-cognate amino acids. 100 ng of purified enzyme was used in each assay. The values of mean and standard deviation were calculated based on three replicates.

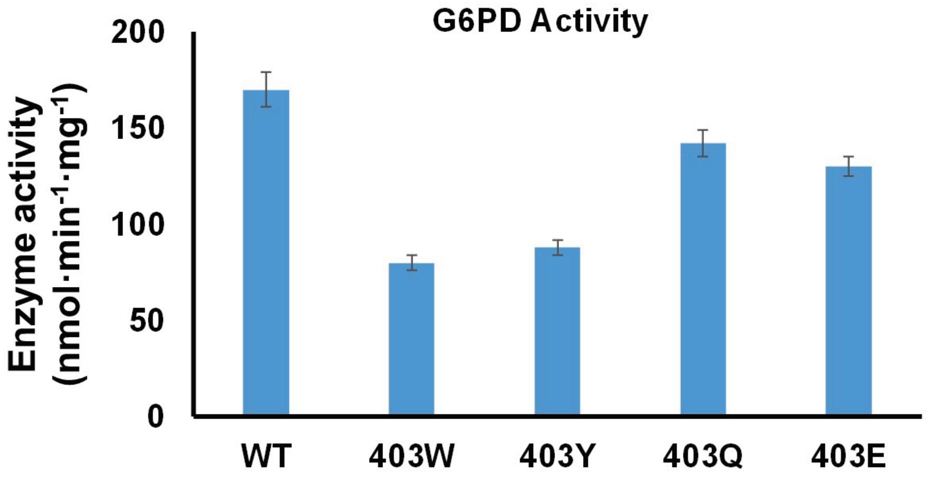

Figure 29. CD spectra of G6PD and its variant. Scanning was performed from 190 nm to 250 nm with a 60 nm/min speed five times for each sample and the average was plotted.

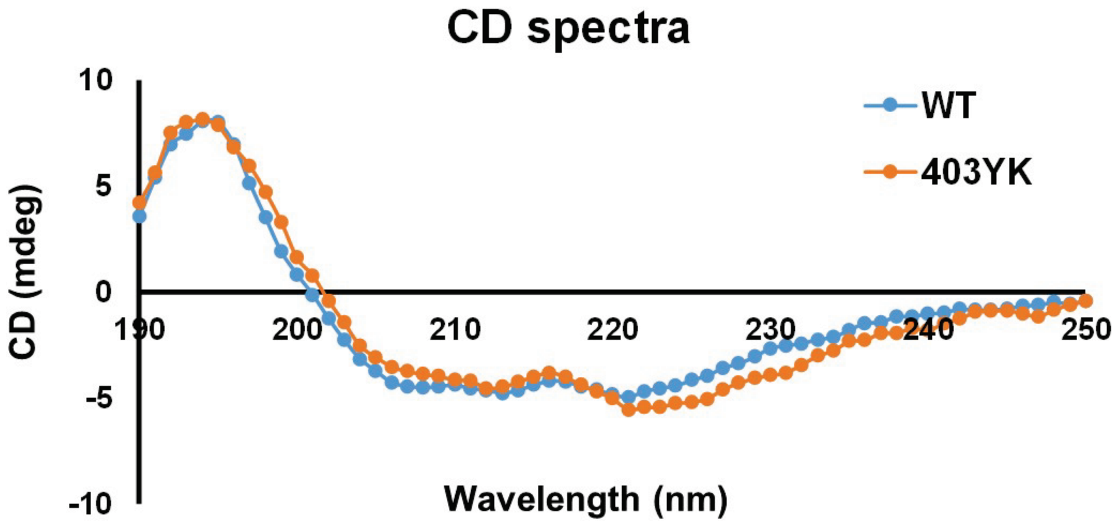

Figure S30. LC-MS/MS analysis of PKM2-62AcK expressed in BL21(DE3) cells. The tandem mass spectrum of the peptide (residues 57-66) SVETLKEMIK from purified PKM2-62AcK. K^{AC} denotes AcK incorporation. The partial sequence of the peptide containing the AcK can be read from the annotated a, b, or y ion series. Matched peaks are in red.

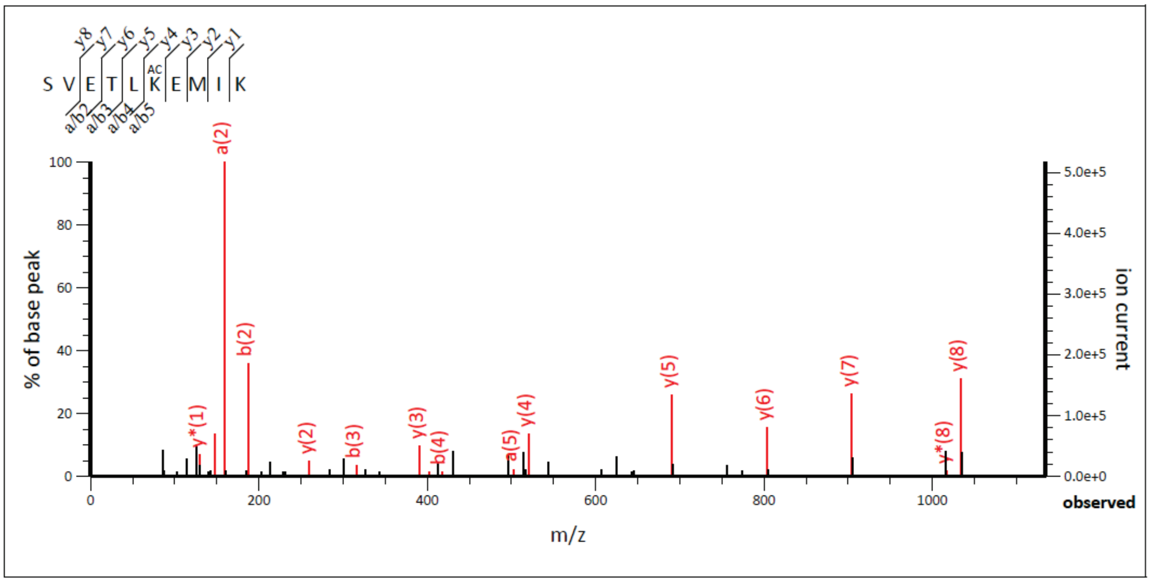

#	a	a++	a*	a*++	b	b++	b*	b*++	Seq.	y	y++	y*	y*++	#
1	60.0444	30.5258			88.0393	44.5233			S					10
2	**159.1128**	80.0600			**187.1077**	94.0575			V	1132.6282	566.8177	1115.6017	558.3045	9
3	288.1554	144.5813			**316.1503**	158.5788			E	**1033.5598**	517.2835	**1016.5333**	508.7703	8
4	389.2031	195.1052			**417.1980**	209.1026			T	**904.5172**	452.7622	887.4907	444.2490	7
5	**502.2871**	251.6472			530.2821	265.6447			L	**803.4695**	**402.2384**	786.4430	393.7251	6
6	672.3927	336.7000	655.3661	328.1867	700.3876	350.6974	683.3610	342.1842	K	**690.3855**	345.6964	673.3589	337.1831	5
7	801.4353	401.2213	784.4087	392.7080	829.4302	415.2187	812.4036	406.7055	E	**520.2799**	260.6436	503.2534	252.1303	4
8	932.4757	466.7415	915.4492	458.2282	960.4707	480.7390	943.4441	472.2257	M	**391.2374**	196.1223	374.2108	187.6090	3
9	1045.5598	523.2835	1028.5333	514.7703	1073.5547	537.2810	1056.5282	528.7677	I	**260.1969**	130.6021	243.1703	122.0888	2
10									K	**147.1128**	74.0600	**130.0863**	65.5468	1

Figure S31. LC-MS/MS analysis of PKM2-66AcK expressed in BL21(DE3) cells. The tandem mass spectrum of the peptide (residues 63-73) EMIKSGMNVAR from purified PKM2-66AcK. K^{AC} denotes AcK incorporation. The partial sequence of the peptide containing the AcK can be read from the annotated a, b, or y ion series. Matched peaks are in red.

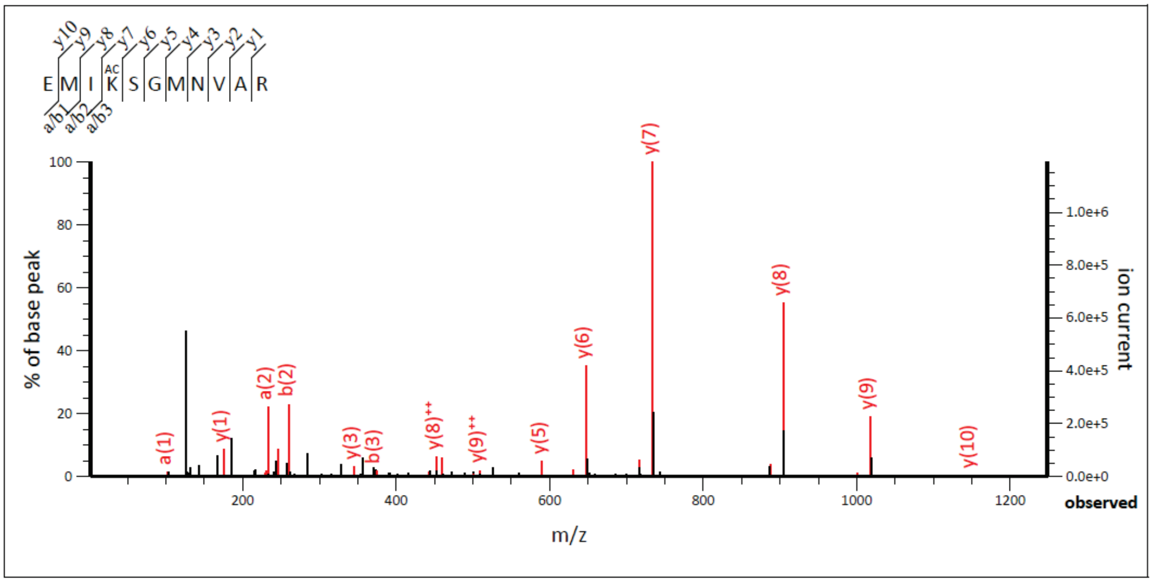

#	a	a^{++}	a*	a^{*++}	b	b^{++}	b*	b^{*++}	Seq.	y	y^{++}	y*	y^{*++}	#
1	**102.0550**	51.5311			130.0499	65.5286			E					11
2	**233.0954**	117.0514			**261.0904**	131.0488			M	**1148.5915**	574.7994	1131.5649	566.2861	10
3	346.1795	173.5934			**374.1744**	187.5908			I	**1017.5510**	**509.2791**	**1000.5244**	**500.7659**	9
4	516.2850	258.6462	499.2585	250.1329	544.2799	272.6436	527.2534	264.1303	K	**904.4669**	**452.7371**	**887.4404**	**444.2238**	8
5	603.3171	302.1622	586.2905	293.6489	631.3120	316.1596	614.2854	307.6464	S	**734.3614**	367.6843	**717.3348**	359.1711	7
6	660.3385	330.6729	643.3120	322.1596	688.3334	344.6704	671.3069	336.1571	G	**647.3294**	324.1683	**630.3028**	315.6550	6
7	791.3790	396.1931	774.3525	387.6799	819.3739	410.1906	802.3474	401.6773	M	**590.3079**	295.6576	573.2813	287.1443	5
8	905.4219	453.2146	888.3954	444.7013	933.4169	467.2121	916.3903	458.6988	N	**459.2674**	**230.1373**	**442.2409**	221.6241	4
9	1004.4903	502.7488	987.4638	494.2355	1032.4853	516.7463	1015.4587	508.2330	V	**345.2245**	173.1159	328.1979	164.6026	3
10	1075.5275	538.2674	1058.5009	529.7541	1103.5224	552.2648	1086.4958	543.7516	A	**246.1561**	123.5817	**229.1295**	115.0684	2
11									R	**175.1190**	88.0631	**158.0924**	79.5498	1

Figure S32. LC-MS/MS analysis of G6PD-403AcK expressed in BL21(DE3) cells. The tandem mass spectrum of the peptide (residues 394-407) VQPNEAVYTKMMTK from purified G6PD-403AcK. K^{AC} denotes AcK incorporation. The partial sequence of the peptide containing the AcK can be read from the annotated a, b, or y ion series. Matched peaks are in red.

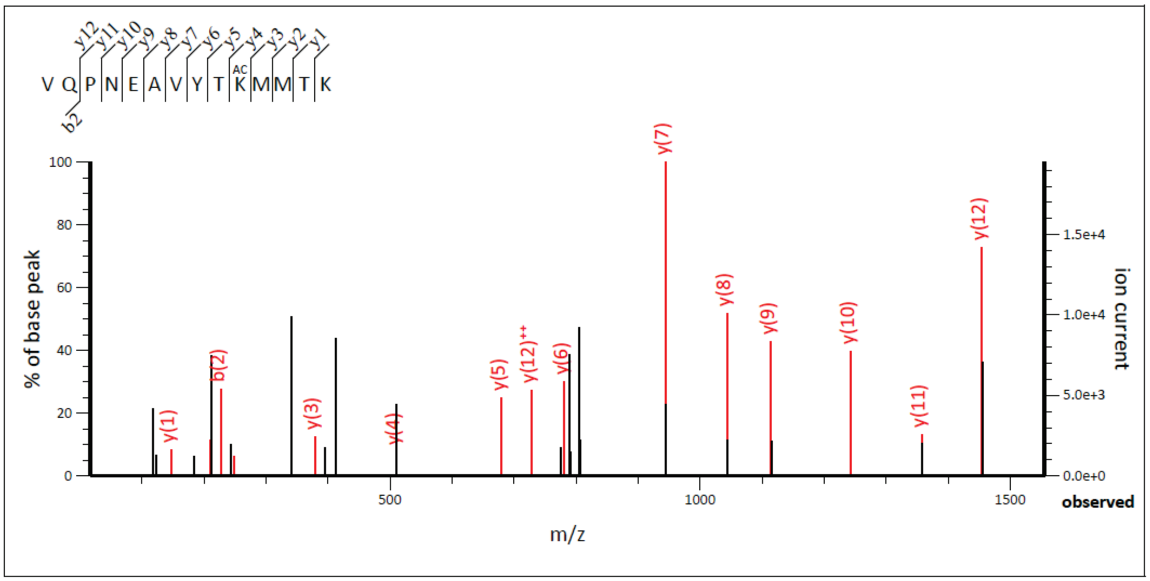

#	a	a++	a*	a*++	b	b++	b*	b*++	Seq.	y	y++	y*	y*++	#
1	72.0808	36.5440			100.0757	50.5415			V					14
2	200.1394	100.5733	183.1128	92.0600	**228.1343**	114.5708	**211.1077**	106.0575	Q	1582.7604	791.8838	1565.7338	783.3706	13
3	297.1921	149.0997	280.1656	140.5864	325.1870	163.0972	308.1605	154.5839	P	**1454.7018**	**727.8545**	1437.6753	719.3413	12
4	411.2350	206.1212	394.2085	197.6079	439.2300	220.1186	422.2034	211.6053	N	**1357.6490**	679.3282	1340.6225	670.8149	11
5	540.2776	270.6425	523.2511	262.1292	568.2726	284.6399	551.2460	276.1266	E	**1243.6061**	622.3067	1226.5796	613.7934	10
6	611.3148	306.1610	594.2882	297.6477	639.3097	320.1585	622.2831	311.6452	A	**1114.5635**	557.7854	1097.5370	549.2721	9
7	710.3832	355.6952	693.3566	347.1819	738.3781	369.6927	721.3515	361.1794	V	**1043.5264**	522.2668	1026.4999	513.7536	8
8	873.4465	437.2269	856.4199	428.7136	901.4414	451.2243	884.4149	442.7111	Y	**944.4580**	472.7326	927.4314	464.2194	7
9	974.4942	487.7507	957.4676	479.2375	1002.4891	501.7482	985.4625	493.2349	T	**781.3947**	391.2010	764.3681	382.6877	6
10	1144.5997	572.8035	1127.5732	564.2902	1172.5946	586.8009	1155.5681	578.2877	K	**680.3470**	340.6771	663.3204	332.1639	5
11	1275.6402	638.3237	1258.6136	629.8105	1303.6351	652.3212	1286.6086	643.8079	M	**510.2415**	255.6244	493.2149	247.1111	4
12	1406.6807	703.8440	1389.6541	695.3307	1434.6756	717.8414	1417.6490	709.3282	M	**379.2010**	190.1041	362.1744	181.5908	3
13	1507.7284	754.3678	1490.7018	745.8545	1535.7233	768.3653	1518.6967	759.8520	T	**248.1605**	124.5839	231.1339	116.0706	2
14									K	**147.1128**	74.0600	130.0863	65.5468	1

Figure S33. Full images of western blotting for PKM2 expressed in 293T cells. Western protein markers were added in the leftmost lanes.

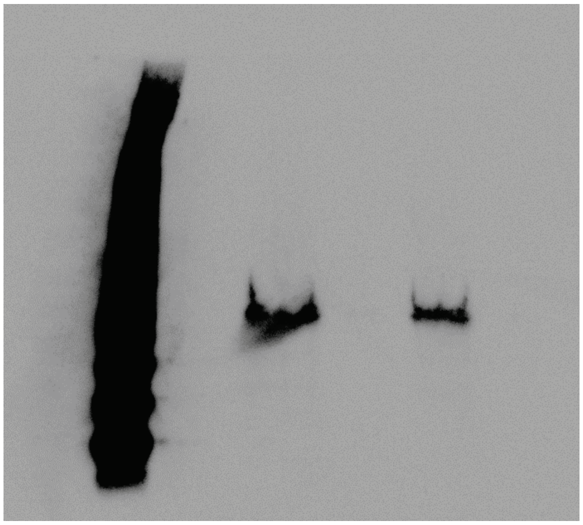

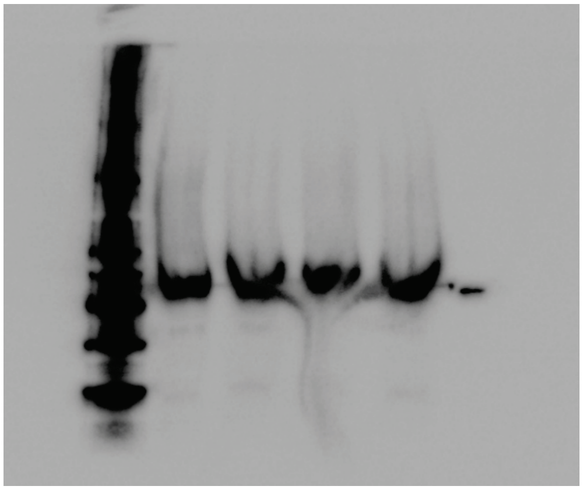

Figure S34. LC-MS/MS analysis of PKM2-62-ValK expressed in HEK293T cells. The tandem mass spectrum of the peptide (residues 57-66) SVETLKEMIK from purified PKM2-62-ValK. K^{VA} denotes Val-K incorporation. M^{OX} denotes oxidation of methionine. The partial sequence of the peptide containing the Val-K can be read from the annotated a, b, or y ion series. Matched peaks are in red.

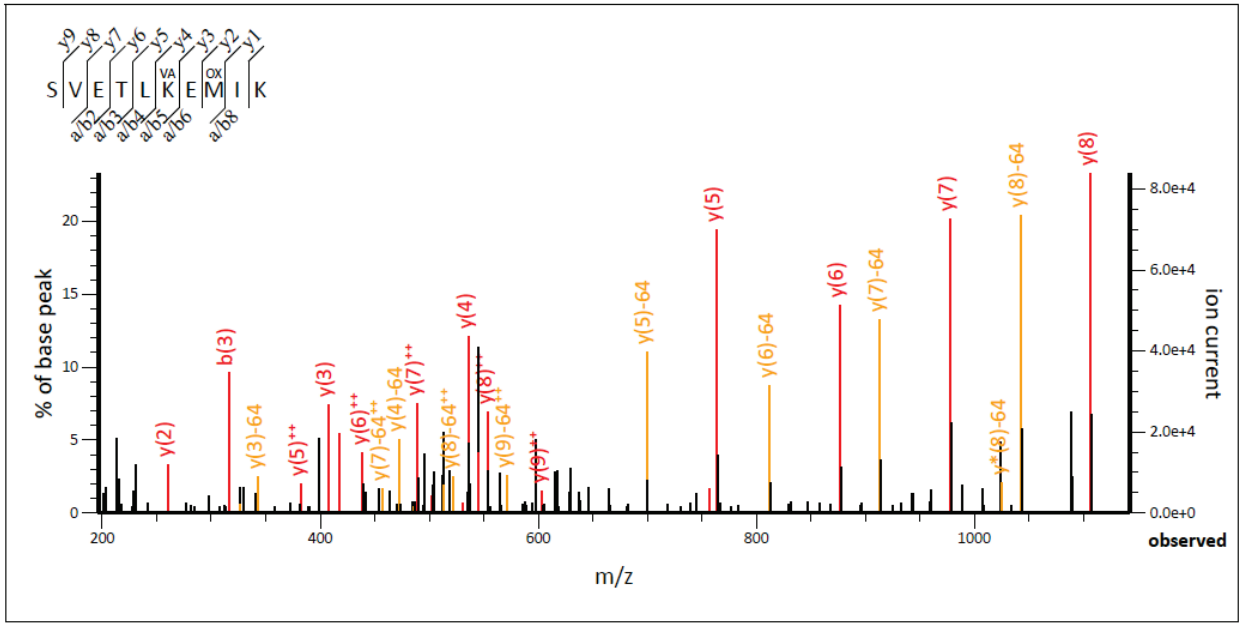

#	a	a^{++}	a*	a^{*++}	b	b^{++}	b*	b^{*++}	Seq.	y	y^{++}	y*	y^{*++}	#
1	60.0444	30.5258			88.0393	44.5233			S					10
2	**159.1128**	80.0600			**187.1077**	94.0575			V	1205.6810	**603.3441**	1188.6544	594.8309	9
3	288.1554	144.5813			**316.1503**	158.5788			E	**1106.6126**	**553.8099**	1089.5860	**545.2967**	8
4	389.2031	195.1052			**417.1980**	209.1026			T	**977.5700**	**489.2886**	960.5434	480.7754	7
5	**502.2871**	251.6472			**530.2821**	265.6447			L	**876.5223**	**438.7648**	859.4958	430.2515	6
6	729.4505	365.2289	712.4240	356.7156	**757.4454**	379.2264	740.4189	370.7131	K	**763.4382**	**382.2228**	746.4117	373.7095	5
7	858.4931	429.7502	841.4666	421.2369	886.4880	443.7477	869.4615	435.2344	E	**536.2749**	268.6411	519.2483	260.1278	4
8	1005.5285	503.2679	988.5020	494.7546	1033.5234	517.2654	1016.4969	508.7521	M	**407.2323**	204.1198	390.2057	195.6065	3
9	1118.6126	559.8099	1101.5860	551.2967	1146.6075	573.8074	1129.5809	565.2941	I	**260.1969**	130.6021	243.1703	122.0888	2
10									K	**147.1128**	74.0600	**130.0863**	65.5468	1

Figure S35. The structure of PylRS and additional mutation sites. The structures of pyrrolysine and the amino acid binding pocket of WT PylRS with Pyl-AMP (PDB ID: 2Q7H). Additional mutation sites to generate the library of PylRS variants were marked with red color.

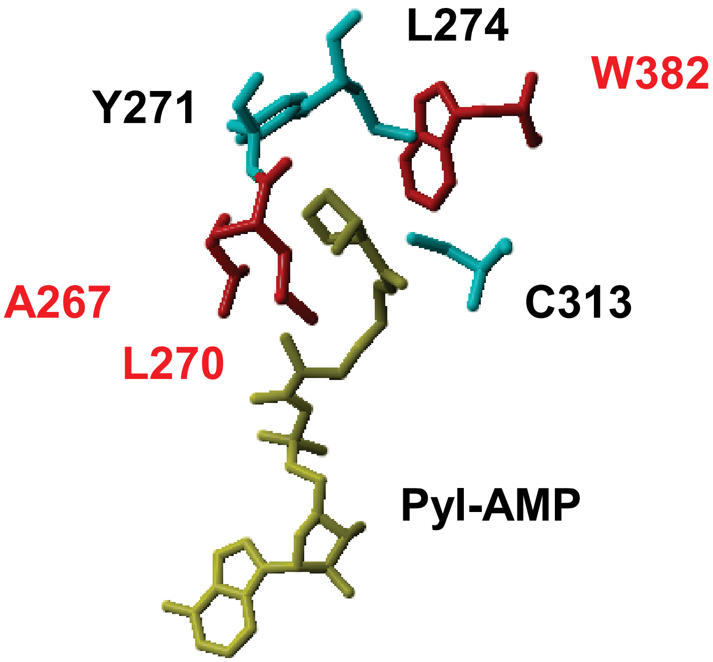

Figure S36. The structure of *Pseudomonas aeruginosa* aspartyl-tRNA synthetase with tRNA. PDB ID: 4wj4. AspRS is colored with blue, and tRNA is colored with purple.

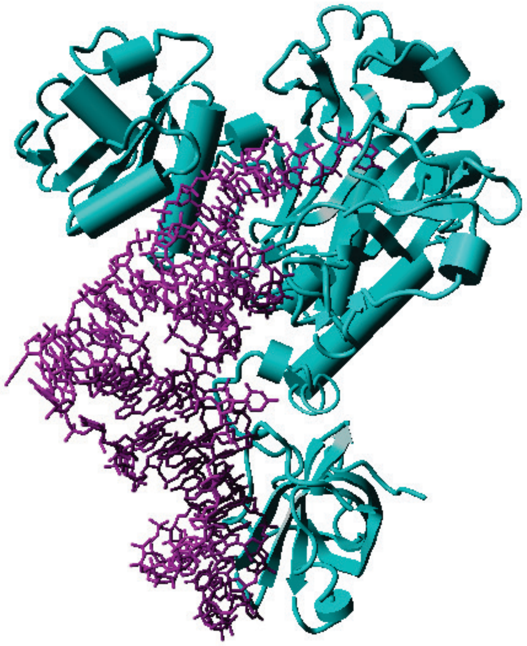

Supplement: Supplementary file 4 [file ja6c03157_si_004.pdf]
